# Mechanical Properties of Nylon Harp Strings

**DOI:** 10.3390/ma10050497

**Published:** 2017-05-04

**Authors:** Nicolas Lynch-Aird, Jim Woodhouse

**Affiliations:** 1 The Old Forge, Burnt House Lane, Battisford, Suffolk IP14 2ND, UK; 2 Cambridge University Engineering Department, Trumpington St, Cambridge CB2 1PZ, UK; jw12@cam.ac.uk

**Keywords:** polymer, nylon, viscoelasticity, string, tuning, harp

## Abstract

Monofilament nylon strings with a range of diameters, commercially marketed as harp strings, have been tested to establish their long-term mechanical properties. Once a string had settled into a desired stress state, the Young’s modulus was measured by a variety of methods that probe different time-scales. The modulus was found to be a strong function of testing frequency and also a strong function of stress. Strings were also subjected to cyclical variations of temperature, allowing various thermal properties to be measured: the coefficient of linear thermal expansion and the thermal sensitivities of tuning, Young’s modulus and density. The results revealed that the particular strings tested are divided into two groups with very different properties: stress-strain behaviour differing by a factor of two and some parametric sensitivities even having the opposite sign. Within each group, correlation studies allowed simple functional fits to be found to the key properties, which have the potential to be used in automated tuning systems for harp strings.

## 1. Introduction

It is well known that the properties of musical instrument strings vary significantly with time, temperature and humidity, requiring players to retune their instruments frequently. The tuning of nylon and natural gut strings in particular can vary noticeably during the course of a performance. For instruments with a fingerboard, the player can make some correction by changing the effective length of the string being played, but for open string instruments, and the harp in particular, the player can really only wait until the next break in the music to retune an errant string.

Automated string tuning systems have been developed [1,2], but these generally require the instrument to be switched into a specific tuning mode, with the string being plucked or struck in order to generate a tone that can be compared with the required musical pitch. A motor is then used to adjust the string tension as necessary. Even if such a system were adapted for use during a performance, it would not help with the situation where the string is already noticeably out of tune when it next comes to be played. Ideally, a tuning system would make pre-emptive adjustments to maintain the string’s pitch while it is not being played.

Such a system would somehow need to determine the pitch of an unplayed string or else predict how the string will respond to changes in temperature and humidity and, especially for a new string, simply the passing of time. In either case, determining the scaling factors to convert a given tuning error into the required adjustment, with sufficient accuracy that the tuning control system is both stable and able to make the necessary corrections with the minimum number of iterations, requires an understanding of the string’s Young’s modulus. More specifically, the tensile “tangent modulus” (the stress/strain ratio for small perturbations) of the string at its expected operating point is needed.

The fundamental frequency $f_{1}$ of a stretched flexible string is given to a good approximation by the well-established equation [3]:

(1) $$f_{1}=\frac{1}{2L_{V}}\sqrt{\frac{F}{\mu }}=\frac{1}{2L_{V}}\sqrt{\frac{\sigma }{\rho }}$$

where *F* is the string tension, μ is its linear density, $L_{V}$ is the vibrating length, ρ is the material density and σ=F/A is the stress, *A* being the cross-sectional area. The string tension is determined by two factors: the string’s tensile stiffness and the amount by which it has been stretched. The tension may be affected by changes in temperature and humidity, through variation of the string’s Young’s modulus and also through the effects of thermal expansion. The linear density of the string is affected by changes to the mass within the active vibrating section of string, caused by the initial tuning of the string and subsequent adjustments, and also by changes in temperature and humidity.

There is a limited amount of published information regarding the Young’s modulus of nylon musical strings [4,5], but there is almost nothing in the existing literature quantifying how such strings behave in response to changes in temperature and humidity. Some information is available for bulk nylon [6,7,8], but as will be shown here, stretched musical strings do not behave in the same way. Nylon is not greatly affected by changes in humidity [6,7], and the emphasis here is therefore on the effects of temperature changes.

This paper presents some results from an investigation into the mechanical behaviour of nylon harp strings. The work included investigations of the time-dependent creep response of such strings, but this paper concerns only the relatively steady behaviour after the initial phase of rapid creep has settled down. This is the regime of most direct interest to the musician, who would normally be using well-settled strings during a concert performance. The effects of the string’s stretching on its density and working tension are first examined, followed by a study of the Young’s modulus, all at a base temperature of 20 °C. This is followed by an examination of how the string’s fundamental frequency, tension and linear density are affected by changes in temperature: information that would be needed in any pre-emptive tuning system that monitored the temperature and tried to compensate the string tuning. As well as reporting the observed behaviour, attempts are made via correlation studies to identify the key dependencies on the string dimensions, material density and the applied stress and temperature. The bulk of the measurements were taken at fairly consistent humidity levels around 60% RH (relative humidity) at 20 °C, but some findings at much lower humidity levels are also included.

Nylon, being a polymer, will exhibit viscoelastic stress-strain behaviour. Even within the small-strain regime of linearised theory, this means that the Young’s modulus will not be a simple number. As with any elastic modulus, it can only be properly defined in the frequency domain, where it will be a complex number, which will vary with frequency (see for example, Bland [9]). The complexity reflects the fact that there will be a phase difference between the modulations of stress and strain, representing energy dissipation.

Variation of Young’s modulus with frequency is mathematically inevitable on grounds of causality (see for example, Crandall [10]), but in the case of nylon musical strings, the basic phenomenon is intuitively familiar. When a new string is first fitted it will be tensioned to give the correct pitch, but after a short time it will have exhibited some creep so that, in musical terms, it will have gone flat. If the string is repeatedly re-tuned to the correct pitch, this creep phenomenon usually runs its course over a time-scale of a few days. A musician would then describe the string as “settled”, only requiring relatively minor tuning adjustments.

Translated into the language of Young’s modulus, this means that if adjustments were made very slowly compared to the creep time-scale, in other words at a very low frequency, a relatively low value of Young’s modulus would be measured (because it includes the effects of creep). On the other hand, if changes are made on a time-scale that is short compared to the creep time-scale (in other words, at a higher frequency), a higher value of Young’s modulus will be obtained. In the tests to be described here, Young’s modulus was measured over three very different time-scales: order of weeks, order of minutes and order of milliseconds. All three scales are relevant to the musical application.

## 2. Theoretical Background

A gauge length *L* of string will be stretched by a time-varying tension F(t), resulting in a length x(t) being pulled beyond the gauge region, which is assumed fixed: *x* will be called the “length adjustment”. The cross-sectional area of the string A(t) will be assumed to remain spatially uniform at all times: “necking” will not be considered. For very small strains, the density ρ of the material would be expected to reduce with strain: if Poisson’s ratio is denoted ν, then:

(2) $$\rho \approx \rho_{0}\left[1-\left(1-2\nu \right)\epsilon \right]$$

where $\rho_{0}$ is the initial density and ϵ is the tensile strain. For a typical value of Poisson’s ratio of around 0.35, this suggests:

(3) $$\rho \approx \rho_{0}\left[1-0.3\epsilon \right].$$

However, in the tests to be reported here, significant creep deformation will be seen, and the strains can become quite large (up to 20%). Experience from other areas of plasticity then leads to an expectation that after some initial reduction in density following Equation (3), the density will tend to become constant at larger strains. This expectation will be confirmed by direct measurement in Section 4.

There is then a simple relation between the length adjustment and the area. For a small increase of length adjustment to x+δx resulting from a tension change to F+δF, suppose the area changes to A+δA. Equating two forms of the volume lost by the gauge length,

(4) LδA=-Aδx

so that by integration:

(5) $$A=A_{0}e^{-x/L}$$

The strain within an element of string that remains within the gauge length can also be related to *A* by the constant-density assumption. If an element of initial length *h* has stretched to length h+δh, then:

(6) $$hA_{0}\approx \left(h+\delta h\right)A=\left(h+\delta h\right)A_{0}e^{-x/L}$$

so that the strain is given by:

(7) $$\epsilon =\delta h/h\approx e^{x/L}-1\approx x/L$$

where the final approximation is valid when x≪L.

Next, consider a string at base temperature $T_{0}$, after the initial rapid creep phase has settled (see Section 3.4 for how this was achieved in the experiments). The string is now subjected to a small change in temperature with no change of length. The main concern behind this work is the effect on the tuning of the string. From Equation (1), taking logarithms and differentiating gives two alternative expressions for the temperature sensitivity of the fundamental frequency:

(8) $$\frac{1}{f_{0}}{\left.\frac{df}{dT}\right|}_{L}=\frac{1}{2F_{0}}\frac{dF}{dT}-\frac{1}{2\mu_{0}}\frac{d\mu }{dT}=\frac{1}{2\sigma_{0}}\frac{d\sigma }{dT}-\frac{1}{2\rho_{0}}\frac{d\rho }{dT}$$

All quantities with the subscript 0 refer to values at the base temperature $T_{0}$, and to avoid confusion, the subscript 1 from the fundamental frequency is omitted here. Tuning deviations are conveniently expressed in cents (¢), there being 1200 ¢ in an octave [11]:

(9) $$\delta f_{¢}=1200\log_{2}\left(\frac{f}{f_{0}}\right)\;¢$$

where *f* and $f_{0}$ are the actual and desired frequencies, respectively. Hence, the thermal tuning sensitivity of a string held at constant length would be expected to be:

(10) $${\left.\frac{df_{¢}}{dT}\right|}_{L}\approx K\left(\frac{1}{F_{0}}\frac{dF}{dT}+\psi \right)\;¢/{}^{\circ}C$$

where $K=600/\ln\left(2\right)$ and ψ describes the first-order thermal variation in the linear density:

(11) $$\psi =-\frac{1}{\mu_{0}}\frac{d\mu }{dT}.$$

The change in temperature will also affect other quantities: the associated variations will be linked by a relation:

(12) F=AEϵ

where *E* is the value of Young’s modulus appropriate to the time-scale of the temperature modulation. Taking logarithms and differentiating again gives an expression linking the various temperature sensitivities:

(13) $$\frac{1}{F_{0}}\frac{dF}{dT}=\frac{1}{A_{0}E_{0}}\frac{d(AE)}{dT}+\frac{1}{\epsilon_{0}}\frac{d\epsilon }{dT}$$

Again, quantities with the subscript 0 refer to values at the base temperature $T_{0}$. The terms in this equation are either directly measurable, or relate to familiar quantities. In particular, note that:

(14) $$-\frac{d\epsilon }{dT}=\alpha$$

where α is the longitudinal coefficient of linear thermal expansion (CLTE), with a positive value corresponding to an expansion of the string for an increase in temperature. Note also that the value of strain used would not be the slow strain of Equation (7), but should be the effective strain corresponding to the measure of tangent modulus being used:

(15) $$\epsilon_{0}=\frac{\sigma_{0}}{E_{0}}=\frac{F_{0}}{A_{0}E_{0}}$$

Combining the above three equations, the thermal variation in the string tension can be given as:

(16) $$\frac{dF}{dT}=\frac{F_{0}}{A_{0}E_{0}}\frac{d(AE)}{dT}-A_{0}E_{0}\alpha$$

The terms AE have been grouped together in Equations (13) and (16) because in the measurements to be described, they cannot reliably be measured separately, as the area changes are very small.

A final theoretical result is needed to support the measurements to be described shortly. One way to characterise Young’s modulus is via the string’s bending stiffness, through its effect on the string’s overtone frequencies [3,12]. As the overtone number *n* increases, the frequency of the overtone $f_{n}$ becomes progressively higher than its ideal harmonic value $nf_{1}$ in the absence of bending stiffness. The theoretical variation takes the form $f_{n}/n\approx a+cn^{2}$. Provided the measured frequencies fit this pattern, best-fitting such a quadratic enables the bending stiffness *B* to be obtained as:

(17) $$B\approx \frac{2cFL_{v}^{2}}{a\pi^{2}}.$$

The Young’s modulus $E_{B}$ can then be obtained using:

(18) $$E_{B}=\frac{B}{I}=\frac{64B}{\pi d^{4}}$$

where $I=\pi d^{4}/64$ is the second moment of area of a uniform circular section of diameter *d*. The thermal sensitivity of the bending stiffness can also be determined, but any thermal changes in *I* cannot be reliably measured, so the measured quantity should be regarded as the thermal sensitivity of $IE_{B}$.

A general point should be emphasised at this point. This paper does not consider any possible influence on perceived pitch of the inharmonicity arising from bending stiffness. Indeed psychoacoustical subtleties of pitch perception are entirely ignored here. When the term ‘frequency’ is used, it always refers to the string’s fundamental frequency $f_{1}$: higher overtones of the string are only ever considered for the single purpose of estimating the bending stiffness as just described.

## 3. Methods and Materials

### 3.1. Test Rig

**Figure 1:**
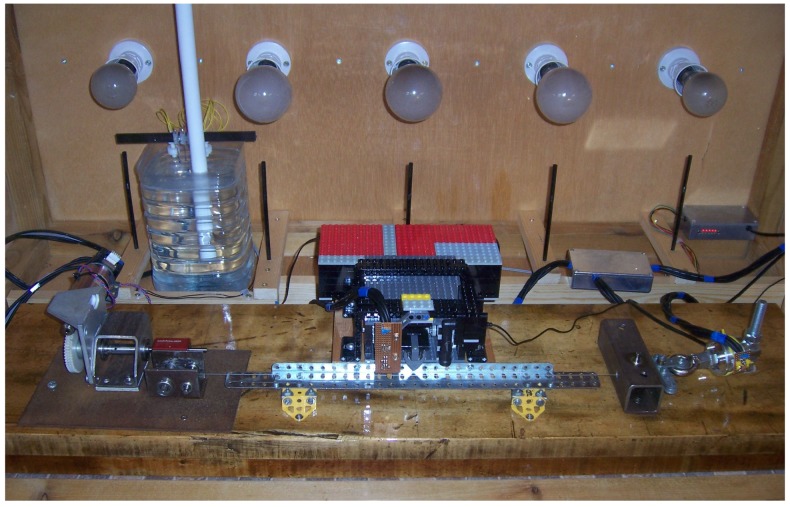
The test rig. The string winder and horizontal bridge pin assembly are mounted on the left of the baseboard, while the vertical bridge pin and load-cell are on the right. The motorised plucker, made from Lego, can be seen in the middle, with the microphone and the temperature and humidity sensor attached. The Meccano strips running behind and below the string provided a heat distribution bar to help even out the temperature along the string. The black plastic drinking straws below the light bulbs acted as light pipes, leading to sensors monitoring the light bulbs.

A test rig (Figure 1) was constructed on which a single string could be stretched horizontally approximately 2 cm above a wooden baseboard. The baseboard was a piece of well-seasoned Canadian rock maple with cross-section 260×68 mm, a little over 1 m in length, and with its grain running along its length. According to the supplier, it was more than 80 years old (being a section of recovered floorboard). A hardwood baseboard was chosen for its relative stability: its CLTE along the grain, and thus approximately parallel to the string, could be expected to be in the range 3.1–4.5 ×$10^{-6}/{}^{\circ}$C [13], which is less than half that of steel [14].

It might be objected that wood is sensitive to changes in humidity. However: (i) the humidity sensitivity is primarily in the cross-grain direction, while the variation in length along the grain is very small [13]; and (ii) the baseboard was thoroughly coated with polyurethane varnish to inhibit moisture penetration. The combination of the varnish layer and the thickness of the board ensured a long time-scale for response to humidity variations, far longer than the 24-h temperature cycle used in the tests to be reported here. As a direct check, a test was carried out using a steel guitar string: the textbook value of CLTE was measured (details not reproduced here).

At one end of the string, a motorised string winder was used for making tuning adjustments. The winding shaft had a diameter of 12.03 mm. For the purposes of calculating string length adjustments, the lengths of string wound onto or off the winding shaft, the turning diameter was taken as the diameter of the winding shaft plus the typical diameter of the unstretched string. In practice, the actual turning diameter would be slightly different since the string would compress a little and also spiral around the winding shaft. These factors could be expected to change slightly as the string was stretched.

Through a combination of an optical rotation sensor mounted on the motor shaft and two stages of worm gear reduction, the rotation of the winding shaft could nominally be resolved to 1 part in 4 million, and in practice, it could be controlled to ±1 part in 80,000, corresponding to a minimum string length adjustment of about 0.5 μm. Tests with a dial gauge showed that the winding shaft radius about its turning axis varied over a range of about 0.06 mm during a full turn, so the absolute accuracy of the string length adjustments was in the range of ±0.03 mm. The total range of the length adjustments applied to the strings corresponded to more than two full turns of the winding shaft, but the range of length adjustments required during each string parameter measurement was typically less than 3 mm (less than 8% of the winding shaft circumference), so the effects of shaft eccentricity were small, and in practice, the accuracy of adjustments was better than ±0.008 mm.

At its other end, the string was attached to a load-cell (Novatech F256EFR0KN 40 kg) for measuring the string tension. The load-cell’s sensitivity and zero offset both varied with temperature, as did the sensitivity of the instrumentation amplifier and analogue-to-digital converter (ADC) used to measure its output. The thermal behaviour of the load-cell and its electronics were therefore characterised using static weights, and compensation functions were devised to produce temperature-corrected tension values. The load-cell was rated up to loads of 400 N, but its electronics were designed to give a full-scale output from the ADC at around 300 N, which was expected to be sufficient to cover the working tensions of the full range of nylon pedal harp strings at their normal operating points. A 10-bit ADC was used together with an oversampling factor of $2^{16}$ providing a nominal resolution of 0.001 N. Tests showed the absolute accuracy of the compensated load-cell and electronics to be within ±0.2 N.

The total string length between the load-cell and winding shaft, the gauge length *L*, was 555 mm. The string was clamped to both the winding shaft and the load-cell to prevent it from slipping. Between these points, the string passed over a pair of bridge pins formed from steel rods 6 mm in diameter. The bridge pins were supported in ball bearings to minimise the frictional resistance when tuning adjustments were made. To further ensure that any tuning adjustments had been propagated along the string, it was always plucked before any measurement was made. The string vibrating length $L_{V}$ between the bridge pins was 500 mm.

The bridge pins were mounted perpendicular to each other (one parallel to the baseboard and one vertical) so that the effective vibrating length of the string was not affected by the direction in which it was plucked. During the initial development of the test rig, with the bridge pins parallel to each other, two distinct resonant peaks could be observed for each of the string’s overtones, corresponding to vibration components parallel and perpendicular to the string’s contact points on the bridge pins [12]. Mounting the bridge pins perpendicular to each other removed this problem.

A motorised plucker, which used a guitar plectrum to pluck the string, was positioned mid-way between the bridge pins, together with a microphone and a digital temperature and humidity sensor. The microphone output was sampled at 96 kHz, and the string pluck responses were recorded for 5 s. The corresponding frequency response therefore spanned a frequency range of 0–48 kHz with a resolution of 0.2 Hz. The resolution of the temperature values was 0.01 °C, with a nominal accuracy of ± 0.3 °C; while the resolution of the humidity values was 0.05% RH, with a nominal accuracy of ±1.8% RH. The plucker was mounted at the mid-point of the string to minimise excitation of the second overtone. This improved the reliability of the automated analysis algorithm in correctly identifying the fundamental frequency in each pluck response.

The whole assembly was contained within a wooden chamber 1.2×0.6×0.6 m with a removable Perspex front panel. Heating was provided using 100-W and 150-W incandescent light bulbs, which could be switched in different combinations via a USB-controlled relay box. Two PC chassis fans were used to provide air circulation within the chamber and a metal heat distribution bar was positioned alongside the string to further even out the temperature around it. A water reservoir was included to maintain the humidity levels within the chamber. A crude degree of humidity control could be achieved by changing the exposed water surface area and by varying the water level within the reservoir. During early tests without the water reservoir, the test chamber dried out significantly, with the humidity falling to about 20% RH. A custom-written program was used to control the test rig, including the selection of the string type and test mode, and the recording of the various measured parameters.

### 3.2. Strings Tested

Thirteen centreless ground (“rectified”) nylon pedal harp strings were tested; eleven from Bowbrand and two from Pirastro. Table 1 lists the strings, with their unstretched diameters and bulk densities, which were measured prior to testing. The string diameters were measured using a manual micrometer with a resolution of 0.01 mm: multiple measurements were taken along the string and averaged to improve the measurement resolution. Masses of known lengths of string were measured by weighing and initial density deduced directly. Most of the strings were weighed on a scale with a resolution of 1 mg, giving density estimates accurate to better than ±1%. However, the Pirastro strings were weighed on a lower-resolution scale with the result that the density error range for string 13 (one of the lightest strings tested) was larger at ±3%.

These strings were part of a larger set, used in a number of studies. When each string was first weighed and measured, it was assigned an identification number, which is shown in the first column of Table 1. Where a string was long enough to provide multiple sections for testing, the string number was supplemented with a suffix letter to differentiate each section. These original identification numbers may seem a little illogical in the context of the present study, but they have been retained for consistency with reports on other tests (for example, [15]). These numbers are used consistently in the summary dataset accompanying this paper and in the much larger comprehensive dataset available at https://doi.org/10.17863/CAM.9018. The note shown for each string in Table 1 indicates its intended usage on the harp and is given using the piano scale. The corresponding string number on the harp string numbering scale is shown in brackets. It is interesting to note that the thicker strings (A3 and below) had a significantly higher density than the thinner strings (A4 and above): this suggests that the two groups of strings had different formulations or were even made from a different base polymer.

### 3.3. Test Protocols

For the first series of tests (using the strings listed in the top seven rows of Table 1), each string was tested at a single target frequency. This fundamental frequency was selected so that the tension would be at or close to the string’s intended operating point. Since the test rig had a fixed vibrating length, while the strings on the harp are all of different lengths, the required target frequency was calculated from the ratio of lengths using Equation (1). The instrument measured to obtain string lengths for this purpose was a Russian-made Elysian Cecilia 46 pedal harp. During the first two tests, with Strings 1 and 6, the water reservoir had not yet been added to the test rig, and the humidity within the test chamber fell to a very low level of around 20–26% RH (at 20 °C). Once the water reservoir was in place, the humidity levels were maintained at around 55–65% RH (at 20 °C) for the remainder of the tests. Consequently, String 1 (A4) was tested only at the lower humidity level, while String 6 (A6) was tested at both humidity levels. All of the other strings listed in Table 1 were tested at the higher humidity level.

The length, diameter and tension of sets of strings designed for use on a harp are scaled by fixed multiples from one octave to the next, with a major design consideration being to ensure a smooth and gradual progression in the string “feel”; defined by Firth as $4F/L_{V}$ [16,17,18]. For the Bowbrand strings, it turned out that the octave multipliers for the string tension and the initial (unstretched) diameter were very close to each other (1.49 and 1.41, respectively), with the result that there was a very nearly linear relationship between these two parameters, at the string operating points, across the range of string gauges ($r^{2}$ = 0.996). In examining the results of the initial series of string tests, it quickly became clear that the basic material properties, such as Young’s modulus and the various temperature coefficients, were all varying with the string gauge, but correlation studies aimed at trying to identify the underlying dependencies were thwarted by the very strong correlation between the string tension and diameter at the chosen test frequencies.

Further tests were therefore conducted with strings of various gauges, each being tested at several different target frequencies. The set of test frequencies was chosen to encompass the operating point frequencies for the different string gauges, with approximately equal changes in the applied stress between these points. The operating point frequency for string 29 (278 Hz) was not included, as this string was added late in the study after the set of test frequencies had already been established. Each time the target frequency was changed, steps were taken (see below) to ensure that the string had essentially stopped creeping before running a set of measurements to determine the string material properties. The range of test frequencies used was limited for the thinner strings by the string breaking or for the thicker strings by the 300 N design limit in the load-cell electronics.

### 3.4. Initial String Settling

An effective way to monitor the progress of string creep was to plot the string length adjustment (being made to keep the string tuned to the target frequency) against the temperature. Cycles of heating and cooling (either ambient temperature changes or an applied heating cycle) resulted in a loop, and any creep showed up as a gradual drift upwards or downwards of that loop. Using this creep monitoring approach, if a string was tuned directly to a new target frequency, it typically took several weeks to settle to the point where it appeared to have stopped creeping. This is a longer settling time than a musician might describe, because the requirements imposed for laboratory testing are more stringent. During the initial series of tests, with each string tested at a single target frequency, this settling delay was not too restrictive. However, to be able to run the tests using multiple target frequencies within an acceptable time, a technique was developed for rapidly settling the string each time its target frequency was changed. The string was initially over-tuned above the target frequency to accelerate the creep rate. The trick was then to bring the frequency back to the required value before the string had crept too far. It was typically found that over-tuning the frequency by 6% (approximately one semitone) for about three days worked quite well. Throughout the over-tuning period, the string was automatically retuned every 5 min.

**Figure 2:**
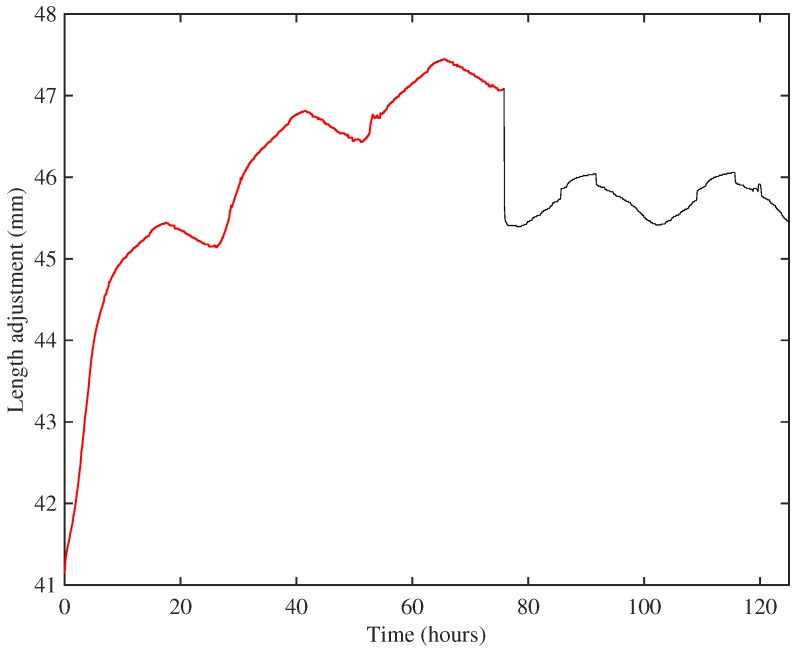
The length adjustment response for String 8 (A6) during the creep settling for the step between 375 Hz and 403 Hz. The string was over-tuned to 427 Hz for about 72 h (red) before being tuned back down to its target frequency of 403 Hz (black).

Simultaneously, a heating and cooling cycle with a 24-h period was applied. To accelerate settling, the maximum temperature limit was set a few degrees higher (typically 45 °C) than that to be used during the string parameter measurements (typically 40 °C). Figure 2 shows the resulting length adjustment response for String 8 (A6) during the creep settling for the step between 375 and 403 Hz. The string was over-tuned to 427 Hz for about 72 h before being tuned back down to its target frequency of 403 Hz. The ripples in the response were due to the tuning adjustments needed to compensate for the applied heating cycle. Using this approach, a string could usually be settled within about a week. Even so, each string was typically on the test rig for a total of several months, and the results reported here were recorded over a period of five years.

### 3.5. Young’s Modulus by Tension Modulation

At each target test frequency, once the string had been settled as described above, tests were run to determine the key string parameters. Foremost among these was Young’s modulus, which was measured as follows while the temperature in the test chamber was varied on a 24-h cycle. The string was initially maintained at its target frequency until the temperature passed through the base temperature of 20 °C. The string tension at this point was taken as the base tension. The tension was then modulated through ±10% in 2% steps (for the early strings tested at a single target frequency) or ±6% in 1.2% steps (for the later strings tested at multiple target frequencies). The modulation range was reduced for the later strings to avoid any risk of further string creep. At each step, the string was adjusted until the tension was within ±0.05 N of the required value. A full tension modulation cycle had the form of a triangular wave and lasted about 30 min. The modulus determined by this method will be denoted $E_{T}$.

It was noticed that in many of the resulting responses for tension versus length adjustment, there were times when the length adjustment seemed to jump. Often this occurred at the top or bottom of the tension modulation cycle, giving the appearance of some form of hysteresis, but at other times, the jumps were part-way along the tension slopes. Closer inspection showed that, as would be expected, the first adjustment made following each change in the target tension was of a fairly consistent size. Depending on the accuracy of the tuning adjustment factor, this first adjustment might then be followed by a series of smaller adjustments to tune the string to the required tension. In some cases, however, the tension did not appear to change following the first adjustment, causing a second adjustment to be made of comparable size to the first one. In other cases, where the first adjustment was too big, the tension then did not change during one or more of the subsequent adjustments being made in the opposite direction, until the first adjustment had been largely negated. It would appear, therefore, that something was occasionally sticking; possibly inside the rolling bearings supporting the bridge pin next to the load cell, where each tension step would be expected to result in a string movement around that bridge pin of only around 5–10 μm. These outsized steps, which could be identified by comparing the ratio of the total length adjustment to the first length adjustment for each tension step, were excluded from the subsequent analysis.

The most direct analysis approach was to calculate the Young’s modulus for each tension step from the corresponding gradient of tension versus length adjustment, using the total string length and its diameter (measured at the end of the test using a manual micrometer). The temperature value for each tension step was simply taken as the average of the temperatures recorded at the start and end of the step. Regression analysis was used to fit linear or quadratic expressions to the resulting data for Young’s modulus versus temperature. This gave estimates for Young’s modulus and its rate of thermal variation at the base temperature. Since the string diameter was only measured once, with no attempt to measure any variation in the diameter with temperature, the measure of thermal variation was more strictly a measure of $\left(1/A_{0}\right)d\left(AE_{T}\right)/dT$ rather than $dE_{T}/dT$ per se. The thicker, higher-density, strings showed a clear curvature in their Young’s modulus versus temperature responses, so in these cases the quadratic expressions were used. In general, the responses for the thinner, lower-density, strings did not show any clear curvature, and so linear fits were used for these strings. More will be said about this curvature in Section 4.2.

As an alternative analysis approach, the tension versus length adjustment gradient was calculated for each step (as before). Linear regression analysis was applied to the steps constituting each rising or falling tension slope, having excluded any outsized steps as described above, to estimate the string stiffness and, hence, Young’s modulus at the base tension (the mid-point of the slope).

**Figure 3:**
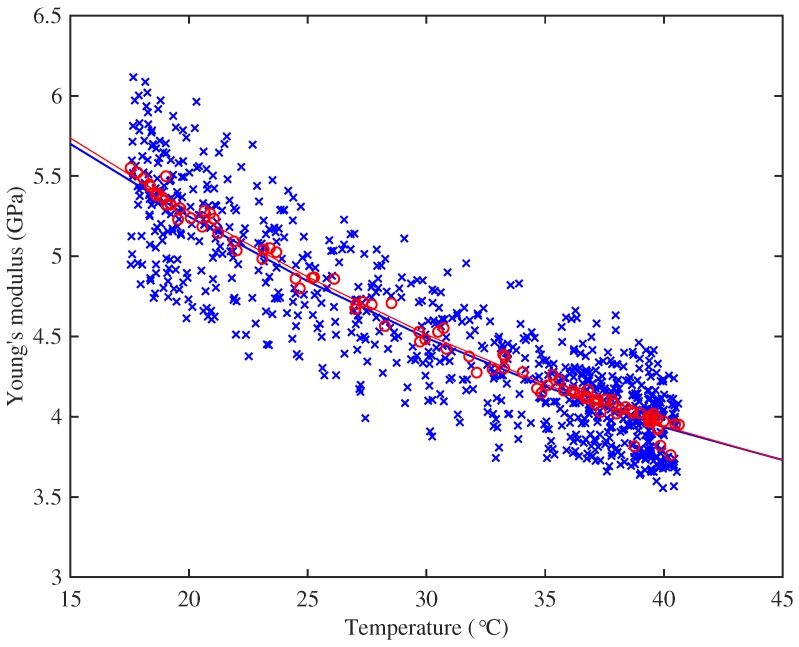
The Young’s modulus versus temperature responses obtained using tension modulation during the testing of String 23a (A3) at 323 Hz. The individual data points (blue crosses) show Young’s modulus values obtained for each individual tension step, after the steps with outsized length adjustments had been excluded. The red circles show the results obtained from the full-slope analysis. The corresponding fitted quadratics are also shown: they are almost identical, so they are hard to distinguish in the plot.

Figure 3 shows the results obtained at one of the test frequencies for the A3 string. As can be seen, there was considerable scatter in the values obtained from the individual tension steps. In general, however, the variations were within the ranges that could be expected, based on the tolerances given above for the tension and length adjustment measurements. The values obtained from the full-slope analysis (red circles) show much less variation, suggesting that much of the scatter in the individual step results could indeed be treated as measurement noise. Reassuringly, quadratic curves fitted to both sets of data gave virtually identical results.

### 3.6. Young’s Modulus from Bending Stiffness

For each of the strings tested at multiple target frequencies, manual pluck responses were recorded at the end of each Young’s modulus test by tension modulation, when the test chamber had anyway to be opened to measure the string diameter. The wire-break method was used [12], in which a thin copper wire is looped once round the string and pulled until it breaks, thereby imparting a clean sharp step function of force to the string. These plucks were applied about 30 mm from one of the bridge pins. This plucking approach was used, in preference to employing the motorised plucker, to provide a sharper pluck with excitation of as many overtones as possible. The pluck responses were analysed to determine the string’s bending stiffness and the corresponding Young’s modulus (see Section 2). The predicted quadratic variation was always observed, similar to that shown in earlier work [12].

For the last four strings tested (Strings 14, 5, 29 and 10b in Table 1), this analysis capability was incorporated into the control program for the test rig, so that the bending stiffness could be measured automatically throughout the string testing. This enabled the variation of $E_{B}$ with temperature to be investigated, as with the tension modulation testing (Figure 3). The responses obtained with the motorised plucker did not have as rich an overtone content as those obtained using the wire-break method, but the ability to analyse a far greater number of pluck responses made up for this. Again, regression analysis could be used to fit lines to the response and provide estimates for Young’s modulus and its rate of thermal variation at the base temperature. As for the tension modulation measurements, the measure of thermal variation was more strictly a measure of $\left(1/I_{0}\right)d\left(IE_{B}\right)/dT$ rather than $dE_{B}/dT$. Results were obtained by keeping the string tuned to its target fundamental frequency until the temperature passed through the base temperature of 20 °C, and then, the string tension was held constant, being re-adjusted as necessary every five minutes, while its temperature was varied on the 24-h cycle already described.

### 3.7. Thermal Sensitivity Tests

Tests were also run in which the string was tuned to its target frequency until the temperature passed through the base temperature of 20 °C, and then, the string length was held constant while the temperature was varied on the 24-h cycle. Frequency measurements were taken every five minutes, and the thermal tuning sensitivity of the string could then be obtained from the gradient of a plot of frequency deviation against temperature.

During these constant-length tests, the linear density of the string was calculated from the string frequency and tension at each measurement point using Equation (1). To explore the tuning variation in more detail, in accordance with Equations (8) and (10), lines and quadratic curves were fitted to the tension and linear density versus temperature responses. As with the results for the Young’s modulus from tension modulation, for the thinner strings, a linear fit generally worked well, but for the thicker strings, a quadratic fit captured the observed behaviour better. These fits gave estimates of the tension $F_{0}$ and linear density of the string $\mu_{0}$ at the base temperature $T_{0}$ and their thermal variations about that point.

Finally, the thermal tension sensitivity results, from the constant length tests, were combined with the results for the thermal variation in Young’s modulus, obtained by tension modulation, to obtain estimates for the longitudinal CLTE of the strings in accordance with Equation (16).

For the earlier strings tested at just a single target frequency, a number of tuning compensation algorithms were also explored: these are considered in Section 5.4.

### 3.8. DMA Tests

To give additional information about material properties, a number of string specimens were also tested in a Dynamical Mechanical Analyser (DMA, Triton Technology TTDMA). Short samples of previously unstretched strings were tested in a clamped 3-point bend configuration with no applied axial stress. The DMA imposed forced vibration on the string sample, inside an environmental chamber that allows temperature to be controlled. Two types of test were used: a frequency sweep at fixed temperature and a temperature sweep at fixed frequency. The temperature sweeps were run at frequencies 1 Hz and 10 Hz, over a temperature range of -20 to +50 °C. The frequency sweeps were run at ambient 20 °C, over the frequency range 0.1–100 Hz. The strings were sufficiently compliant that they were near the limit of capability of the test machine, so that the absolute calibration of measured modulus was not to be trusted. However, as will be described in Section 4.2, a way of estimating a corrected calibration was found.

## 4. Results

### 4.1. Results for Density and Tension

**Figure 4:**
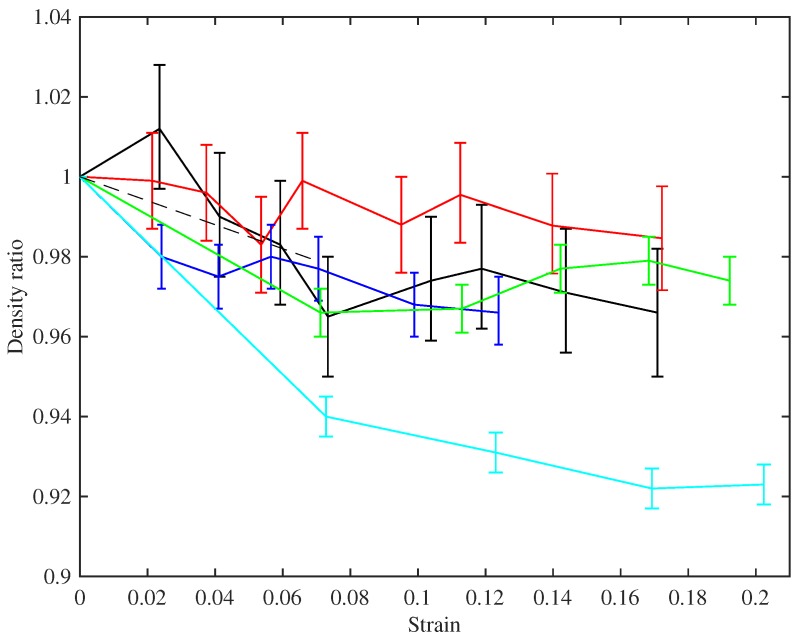
The ratio of the bulk density of the stretched strings to that of the unstretched strings plotted against the strain. The error bars indicate the range of values that could be expected due to the resolution limit of the manual micrometer used to measure the string diameter. Colours identify the different tested strings (Table 1): black (8:A6), red (14:A5), blue (5:A4), green (23a:A3), cyan (29:E3). The dashed line shows the expected reduction in the density ratio for small values of strain.

Figure 4 shows the ratio of the measured bulk density of the stretched strings to their unstretched bulk density, plotted against the strain. The stretched density values were calculated from the linear density at the base temperature, obtained from the constant length tests as described above, and the string diameter, which was measured using a manual micrometer with a resolution of 0.01 mm. The derivation of the strain values is described below in Section 4.2. With the string mounted in the test rig, the diameter could only be measured in a few places and could therefore not be determined as accurately as when the string was first measured prior to testing (when multiple measurements were taken to reduce the measurement error and take better account of any variations along the string length). The error bars in Figure 4 show the range in the density ratio values that could be expected given an error range of ±0.005 mm in the measured string diameter.

The measurements are quite noisy, but the trends follow the expectation described in the theoretical development in Section 2. The string density falls on initial stretching, approximately following the prediction of Equation (3), shown as the dashed line. Within the accuracy of these measurements, the density then remains essentially constant as the strings were stretched further: notice the very narrow range on the vertical axis in the plot. A useful corollary in terms of tuning adjustment is that maintaining a string at constant frequency should have the effect of maintaining it at constant stress, from Equation (1). String 29 is an obvious outlier in this plot, showing a similar qualitative trend, but with density levelling out to around 92% of the original value, rather than something around 97% as seen for the other strings.

**Figure 5:**
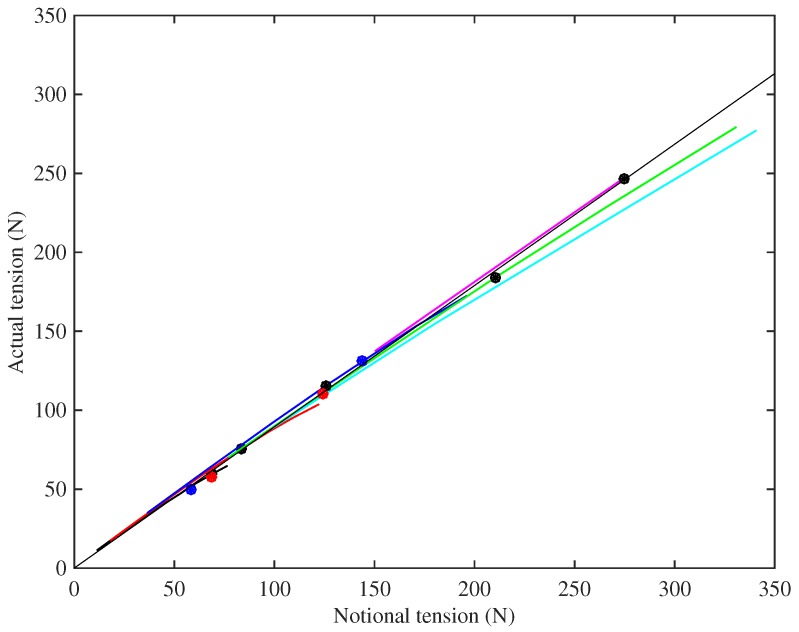
The measured tension of the strings versus the notional tension calculated using the unstretched linear density. Colours identify the different tested strings (Table 1): black (8:A6), red (14:A5), blue (5:A4), green (23a:A3), cyan (29:E3), magenta (10b:A2). Black circles show the results at the expected operating points for the Bowbrand strings tested at the usual humidity levels (around 55–65% RH at 20 °C); blue circles show Pirastro strings; red circles show tests at low humidity (around 20–26% RH at 20 °C).

One important practical problem when designing strings is to predict the working tension once the string has settled. Figure 5 shows the measured tension $F_{0}$ of the different strings, at the base temperature of 20 °C, plotted against the notional tension $F_{N}$ calculated from Equation (1) using the unstretched linear density. Linear regression analysis has been used to fit a line through the operating points (black circles) for the nine Bowbrand strings (excluding String 29) tested at the usual humidity levels (around 55–65% RH at 20 °C). The line was constrained to go through the origin and gave a very good fit ($r^{2}=0.9997$) with a gradient of 0.896. It is reassuring to see that the points for the Pirastro strings (blue circles) and the two Bowbrand strings (red circles) tested at lower humidity levels (around 20–26% RH at 20 °C) also lie close to the fitted line. This suggests that, as a rule of thumb, the working tension of a monofilament nylon musical string will be about 10% lower than the notional value obtained from its unstretched linear density. For the strings tested at multiple target frequencies, it can be seen that the actual tension fell further below the notional values as the tension increased, because the string linear density decreases, primarily as a result of a reduction in its diameter, as the string is stretched.

### 4.2. Results for Young’s Modulus

**Figure 6:**
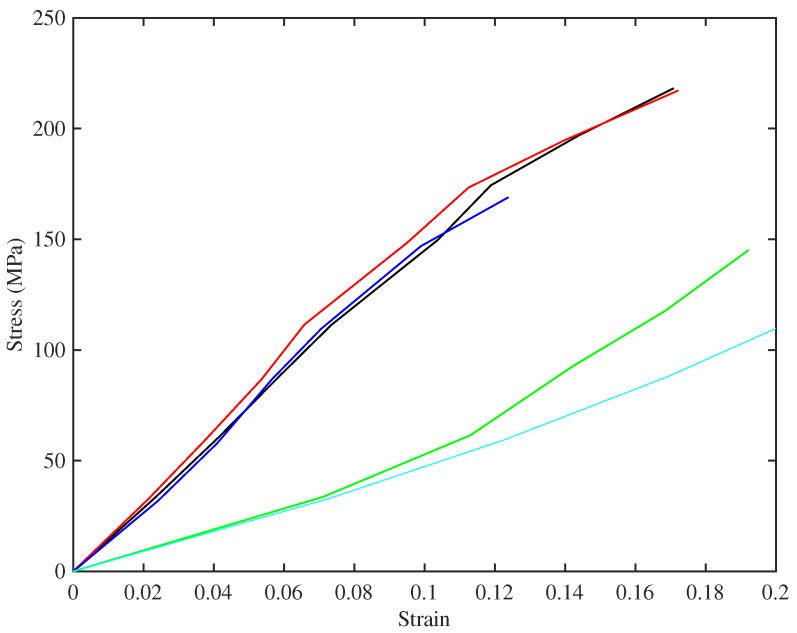
Long-term stress-strain plots at the base temperature of 20 °C, with the strings settled at each point (using the creep-monitoring approach described in Section 3.4). Colours identify the different tested strings (Table 1): black (8:A6), red (14:A5), blue (5:A4), green (23a:A3), cyan (29:E3).

Figure 6 shows the long-term relation between stress and strain for the set of strings tested at multiple target frequencies. Each plotted point here represents the state of a string when it had settled at the chosen stress level (using the creep-monitoring approach described in Section 3.4), at the base temperature of 20°C; so the slopes of the curves give a direct measure of the “slow” Young’s modulus $E_{S}$. When a string was first mounted on the rig, it was hard to define accurately the zero point of length adjustment, because of the details of string take-up onto the winding shaft. As a result, the raw measurements of length adjustment *x* had an unknown offset. However, it is obvious that the correct stress-strain relation should pass through the origin, so to produce this plot, the first three points with non-zero stress for each case have been used to define a quadratic, which was extrapolated back to zero to estimate the offset, which was then subtracted. The strain values used were then calculated from the corrected length adjustments using the exponential form of Equation (7). The process seems to work reliably: the curves shown here all look reassuringly smooth and consistent at low stress levels, and within each group of strings the detailed agreement is excellent. An alternative approach would have been to deduce the strain from the measured linear density, assuming that the total mass remained constant, but that method would not have avoided the difficulties associated with initial take-up and straightening of the string.

It is immediately clear that the strings fall into two distinct groups, with very different behaviour. Referring to Table 1, the same two groups can be identified from the bulk density values, although the disparity is less striking than in the stress-strain behaviour. The strings with lower density produce the upper cluster of lines in the plot: these strings appear generally to show a slight steepening of slope and, hence, increasing modulus, as strain increases up to about 0.07. At higher strain, the slope decreases, indicating reducing modulus. The higher-density strings have a low-strain modulus which is far lower, but on the whole they show increasing slope across the whole range of strain tested here. It seems likely that these groups have some fundamental difference in chemistry and perhaps manufacturing process. Interestingly, one of these strings (Number 29, E3) is the outlier in Figure 4, whereas String 23a (A3), which looks similar here, showed behaviour in Figure 4 that matched the lower-density strings.

**Figure 7:**
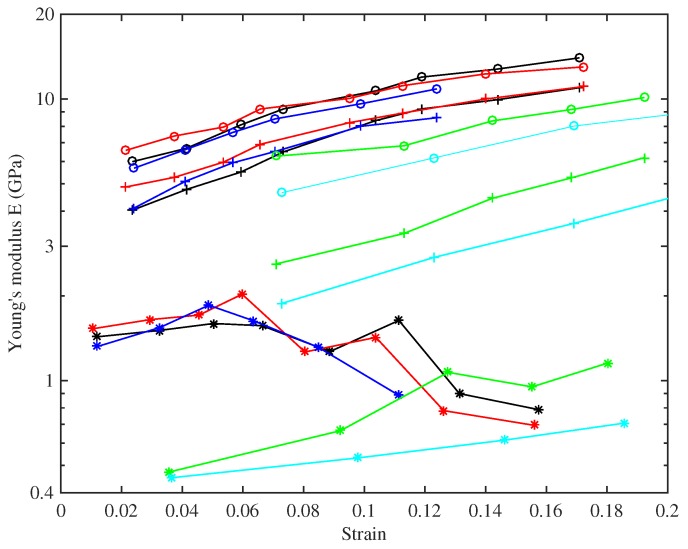
Young’s modulus at the base temperature of 20 °C plotted against the strain, measured by three approaches: $E_{B}$ from bending stiffness (o), $E_{T}$ from modulation of string tension (+) and $E_{S}$ from the slope of the long-term stress-strain plot (*). Colours identify the different tested strings (Table 1): black (8:A6), red (14:A5), blue (5:A4), green (23a:A3), cyan (29:E3).

Figure 7 shows the base temperature Young’s modulus plotted against strain, for all three measurement methods. The slow modulus $E_{S}$ has been calculated from the slopes of the lines in Figure 6, while the measurements of $E_{T}$ and $E_{B}$ were described earlier. This plot reveals quite a complicated pattern of behaviour. For any given string (indicated by colour in the plot), $E_{S}$ has the lowest value; the modulus $E_{T}$ deduced from tension modulation comes next; and the modulus $E_{B}$ deduced from bending stiffness is highest. This pattern is consistent with the intuitive expectation described in the Introduction: Young’s modulus increases as the time-scale of the measurement method reduces. It should be emphasised that all three moduli are relevant to the musical application. The long-term modulus $E_{S}$ relates to the total length correction needed to settle a string to the desired pitch and, hence, determines the final tension shown in Figure 5. The modulus $E_{T}$ measured on a time-scale of minutes is the value needed to perform tuning adjustments, whether manual or automatic. The high-frequency modulus $E_{B}$ influences the sound quality of the plucked string, because the inharmonicity of musical strings resulting from bending stiffness can be audible [19]: it is one reason why different strings plucked in the same way can sound different.

It is clear from Figure 7 that all three moduli also show strong variation with strain. The values of $E_{T}$ and $E_{B}$ both increase monotonically, over a considerable range: $E_{T}$ for String 8 (A6), for example, ranges from around 4 GPa to over 10 GPa. $E_{B}$ for the same string changes by a similar factor, with consistently higher values over the whole tested range. $E_{S}$ for that string shows a different pattern, one already described qualitatively in the discussion of Figure 6: the modulus increases with strain initially, but then declines somewhat for higher strain values. These values are significantly noisier than those of $E_{T}$ and $E_{B}$, because they rely on estimating the slope of the stress-strain plot.

There is another very obvious feature of Figure 7: a contrast in behaviour between the two groups of strings identified from the stress-strain plot. The strings with higher density show considerably lower values of Young’s modulus based on all three measurement methods. The pattern of $E_{S}$ has already been discussed briefly; monotonically increasing with strain, unlike the results for the other group of strings. The results for $E_{T}$ and $E_{B}$ echo this trend. The ratio of $E_{B}$ to $E_{T}$ is bigger for these strings, as is directly visible in this log-scale plot. This may correlate with a more fundamental difference of material constitution, such as a different glass transition temperature. Many polymers show a relation between temperature response and frequency response known as “time-temperature superposition” (see for example, [20]), so a different glass transition temperature might well translate into a different pattern of frequency dependence of elastic moduli.

**Figure 8:**
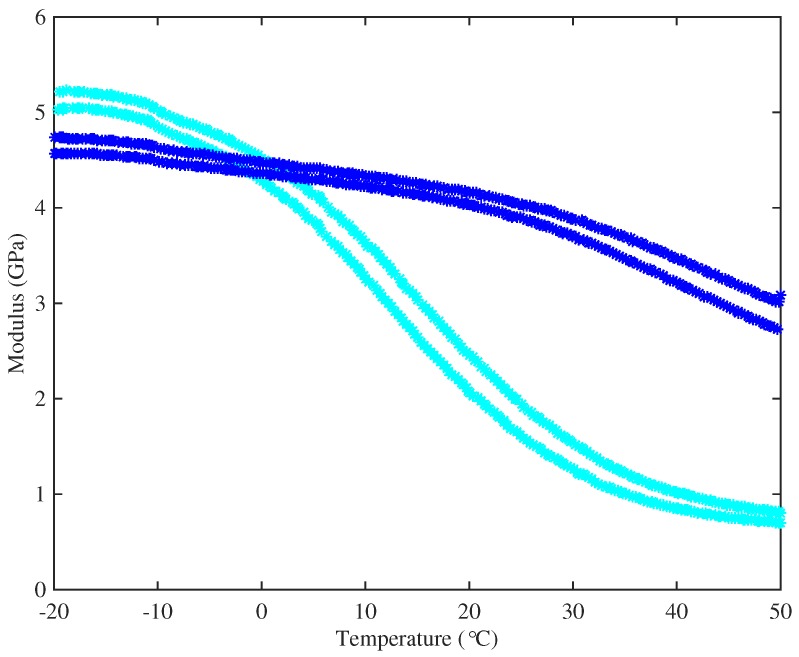
DMA measurements of the real part of Young’s modulus as a function of temperature, for samples of String 5 (A4, blue) and String 29 (E3, cyan). For each string, lines of stars denote measurements at 1 Hz (lower curve) and 10 Hz (upper curve). Tests were done in a clamped three-point bend configuration with zero axial stress.

To explore this conjecture, specimens of strings representative of the two groups were subjected to DMA tests. The main results were very clear. Figure 8 shows the variation of Young’s modulus with temperature for representative examples of strings from the two groups, measured at 1 Hz and 10 Hz: the string with higher density (String 29, E3) has its glass transition range at a significantly lower temperature than the string with lower density (String 5, A4). This figure also shows the expected qualitative effect of frequency: the 10-Hz results are consistently higher than the 1-Hz results for both strings. For the higher-density string, the glass transition straddles the nominal operating temperature of the tests reported here, so that the modulus will have varied significantly during the thermal cycles used. This may indicate the physical mechanism underlying the curvature reported earlier, in the discussion of Section 3.5.

**Figure 9:**
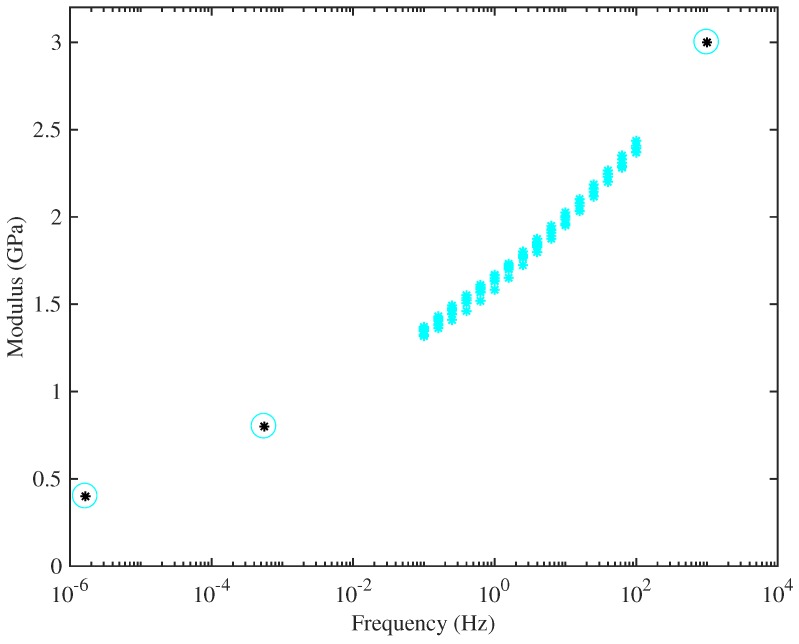
Ambient-temperature measurements of the real part of Young’s modulus of String 29 (E3) as a function of frequency: Dynamical Mechanical Analyser (DMA) tests from the same configuration as in Figure 8 (cyan stars); results from the measurements of $E_{S}$, $E_{T}$ and $E_{B}$, extrapolated to zero axial stress (circles with central black stars). The DMA frequency sweep was repeated several times, indicated by the multiple plotted points.

Figure 9 combines the DMA measurements for String 29 with the values from the other measurement methods already described, deduced by extrapolating the results in Figure 7 to zero strain. Results are plotted on a logarithmic frequency scale. This plot illustrates the approach to correcting the calibration of the DMA tests: a best-fitted scale factor was found, to make the results consistent with the other tests. The plot seems very convincing: with this single scale factor, the results over the entire wide range of frequencies can be seen to fit into a single consistent trend. This suggests that the different measured values of Young’s modulus really do stem mainly from viscoelastic effects, rather than from some shortcoming in the experimental approach.

Returning to the main set of measurements, results from the previously mentioned study of string creep have indicated that the total creep, and hence, strain, depends not simply on the applied stress, but in a more complicated manner on the stretching history of the string and the maximum temperature to which it has been exposed. For the purposes of this study into the long-term behaviour of well-settled strings, it is far from clear a priori whether stress or strain would provide the more useful explanatory variable. In many cases, examining the responses as a function of the applied stress proved to be revealing, especially with regard to identifying the similarities and differences within and between the two groups of strings identified in the stress-strain plots of Figure 6.

**Figure 10:**
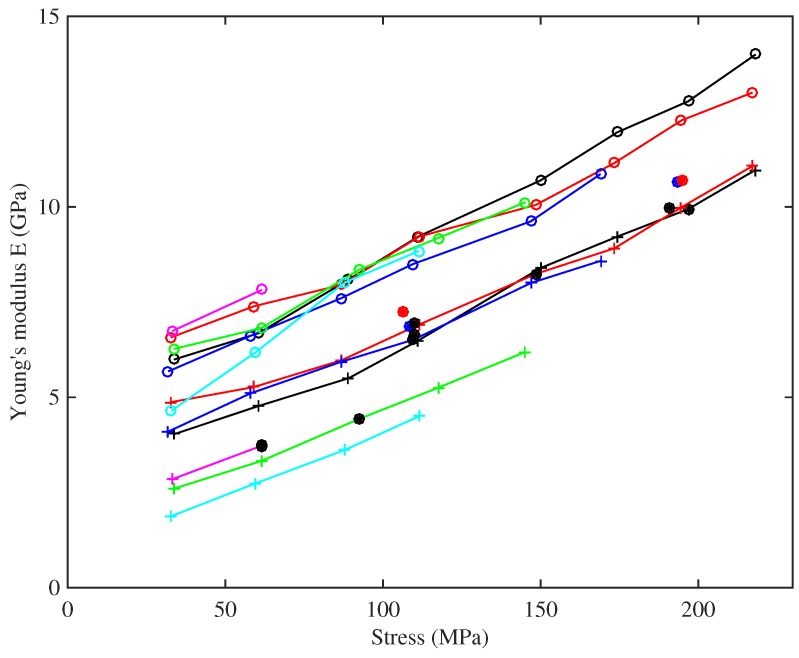
Young’s modulus at the base temperature of 20 °C plotted against the applied stress, measured from bending stiffness (o) and by modulating the string tension (+). Colours identify the different tested strings (Table 1): black (8:A6), red (14:A5), blue (5:A4), green (23a:A3), cyan (29:E3), magenta (10b:A2). Black circles show the results at the expected operating points for the Bowbrand strings tested at the usual humidity levels (around 55–65% RH at 20 °C); blue circles show Pirastro strings; red circles show tests at low humidity (around 20–26% RH at 20 °C).

To illustrate this, Figure 10 shows the results for $E_{T}$ and $E_{B}$ replotted (on a linear scale) against stress. All of the curves can be seen to increase fairly linearly with the stress, and with very similar gradients, for both measures of Young’s modulus and across all of the strings. This indicates a degree of strain hardening, more properly “strain stiffening”, presumably due to straightening of the nylon molecules. The fact that the modulus correlates more clearly with stress than with measured strain can be tentatively explained. Strain in a polymer like nylon results from two mechanisms: straightening of the molecular chains, which is mainly an elastic effect, and slip between adjacent chains as van der Waals bonds break and re-form, which is the mechanism of plastic creep. The results described here were obtained after the creep deformation had had time to equilibrate, and the total measured strain contains a large component of this creep. However, the stiffening effect is associated with the straightening mechanism, and perhaps, stress is a good surrogate measure of this elastic component of the strain. Note that the steady and fairly linear overall stress-strain relation seen in Figure 6 suggests that the progressive reduction in the elastic stretching was being offset by an almost equivalent increase in stretching due to plastic creep.

Figure 10 shows that the observed range of variation in $E_{T}$ also applies to the range of nominal operating points (black circles) for different string gauges: thicker strings operate at low stress, thinner strings at high stress (to the extent that the highest strings on a harp are very prone to spontaneous breakage). This variation would need to be taken into account when designing any automated electronic tuning system. Using the previously available values of around 3–5 GPa for the Young’s modulus of bulk nylon [6] or nylon strings [4,5], when calculating the tuning adjustment scaling factor, would lead to unstable systems in many cases; with successive tuning adjustments overshooting the desired tuning until something broke.

For the results from tension modulation, it can be seen that the two tests run at lower humidity levels (red circles) gave slightly higher Young’s modulus values compared to strings of the same gauge, but the differences were not particularly dramatic given the very large difference in the humidity levels. The A4 Pirastro string, at a stress of about 110 MPa, had a Young’s modulus very similar to that of the Bowbrand A4 strings tested at the usual humidity levels, whereas the thinner A6 Pirastro string, at a stress level of nearly 200 MPa, behaved more like the Bowbrand A6 string tested at low humidity.

**Figure 11:**
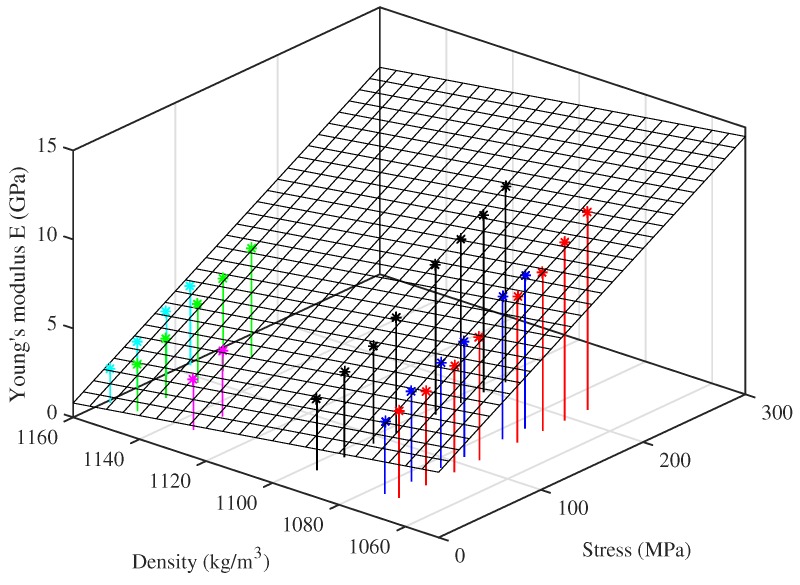
Young’s modulus from tension modulation at the base temperature of 20 °C plotted as a function of stress and unstretched string density. Colours identify the different tested strings (Table 1): black (8:A6), red (14:A5), blue (5:A4), green (23a:A3), cyan (29:E3), magenta (10b:A2).

For the strings tested at multiple target frequencies, the Young’s modulus results derived from the string bending stiffness are quite tightly grouped together, while those obtained by tension modulation are more spaced out. This suggests that there is an additional factor influencing this measure of the Young’s modulus, not directly related to the string tension or stretching. Testing the strings at the same set of frequencies, and hence at approximately the same stress levels, allowed a pattern to be discerned in the Young’s modulus as a function of stress level and unstretched string density, shown in Figure 11. This plot suggests that, for a given stress, $E_{T}$ appears to fall fairly linearly as the string density increases. Presumably the density variations arise from chemical or microstructural differences, which also have an influence on the Young’s modulus.

Motivated by this observation, a curve-fit expression was derived using multiple regression analysis applied to the results from the ten Bowbrand strings tested at the usual humidity levels (around 55–65% RH at 20 °C). The tension-modulation Young’s modulus $E_{T0}$ at 20 °C can be estimated with reasonable accuracy from the notional stress $\sigma_{N}=4\rho_{N}L_{V}^{2}f_{1}^{2}$ (expressed in MPa) and the unstretched bulk density $\rho_{N}$ (expressed in kg/m^3^) by the relation:

(19) $$E_{T0}\approx 32.0+0.0353\sigma_{N}-0.0269\rho_{N}\;\mathrm{GPa}.$$

While the model is not perfect, it is certainly good enough to ensure a stable yet responsive string tuning control system for all of the tested strings. Indeed, an earlier version of this expression, based on fewer measurements and without the density term, was successfully used in the test rig control program for much of the testing.

Returning to Figure 10, it can be seen that the values of $E_{B}$ derived from bending stiffness exhibit a very similar linearly increasing trend with stress, while being consistently higher than those of $E_{T}$. Frequency dependence arising from viscoelasticity has been suggested as the main explanation of this (see Figure 9 and the associated discussion), but it is not the only conceivable reason. Another possible contributory factor is that the stiffness of the strings might not be uniform across their cross-section, which would change the relationship between Young’s modulus and bending stiffness assumed in Equation (18). Bending stiffness is preferentially influenced by the outer layers, whereas for axial stretching, the combined stiffness is a straightforward average over the whole cross-section.

**Figure 12:**
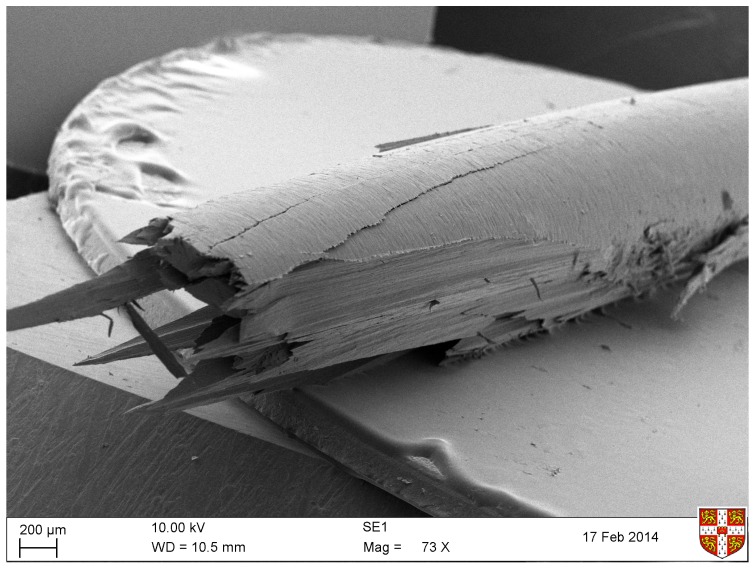
Scanning electron microscope image of a section of the A4 (1.2 mm diameter) Bowbrand centreless ground nylon pedal harp string, which was dipped in liquid nitrogen and then snapped.

Figure 12 shows a scanning electron micrograph of the end of a section of the A4 (1.2 mm diameter) nylon harp string which has been dipped in liquid nitrogen and then snapped. The string appears to have a thin ‘skin’ wrapped transversely around the longitudinal ‘grain’, perhaps as a result of the grinding process used to reduce the string to the required diameter. Tests with thinner and thicker strings showed similar surface layers, and they all appeared to be just a few microns thick. This skin structure is interesting, but if anything, it would be expected to result in an opposite trend to the observation: if the outer layer of the string is less stiff in the axial direction than the core, the bending measurement would tend to give a lower modulus than the tension-modulation test.

### 4.3. Results for Thermal Tuning Sensitivity

**Figure 13:**
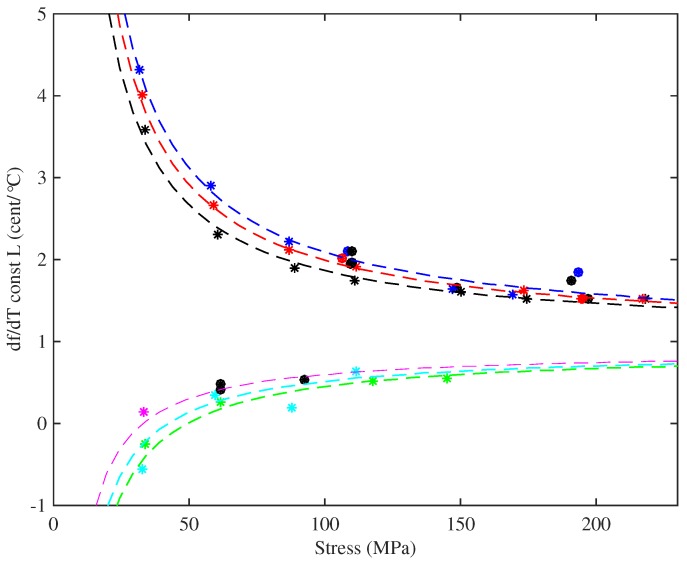
The thermal tuning sensitivity, plotted against the applied stress, for the strings held at constant length with no tuning adjustments. The *y*-axis shows the rate of change of fundamental frequency $f_{1}$ with temperature *T*. Colours identify the different tested strings (Table 1): black (8:A6), red (14:A5), blue (5:A4), green (23a:A3), cyan (29:E3), magenta (10b:A2). Black circles show the results at the expected operating points for the Bowbrand strings tested at the usual humidity levels (around 55–65% RH at 20 °C); blue circles show Pirastro strings; red circles show tests at low humidity (around 20–26% RH at 20 °C). The dashed lines show estimated responses obtained using Equation (20).

Figure 13 shows a quantity of direct significance to the musician: the thermal tuning sensitivity, in ¢/°C, for the strings held at constant length with no tuning adjustments, plotted against the applied stress. The dashed lines show estimated curves, discussed below. In almost every case tested, the string went sharp as the temperature was increased, unless the temperature was taken high enough to cause the string to start creeping again. It is generally accepted that the minimum perceptible change in pitch of a musical note is around 3 ¢, though this varies with the pitch and complexity of the sound [21]. Many of the strings tested varied at rates in excess of 1.5 ¢/°C, so even a modest change in temperature could be expected to lead to noticeable changes in the string pitch. This phenomenon, familiar to harpists, was the original motivation for this study, in the hope of being able to design a tuning compensator to offset the problem. The two Pirastro strings behaved in a similar manner to the Bowbrand strings, and reducing the humidity level did not appear to affect the overall tuning sensitivity.

Figure 13 shows a striking difference between the lower- and higher-density groups of strings, with the higher-density strings showing significantly lower sensitivity to temperature changes. Within each group, however, the variation in thermal tuning sensitivity with stress was consistent. All of the results showed a very strong correlation between the observed thermal tuning sensitivity and the inverse of the applied stress. Referring to the right-hand side of Equation (8), this rather suggests that the rate of change of stress with temperature, of an unadjusted nylon string, is more or less constant, both for a given string and a string of a given material or chemical make-up.

**Figure 14:**
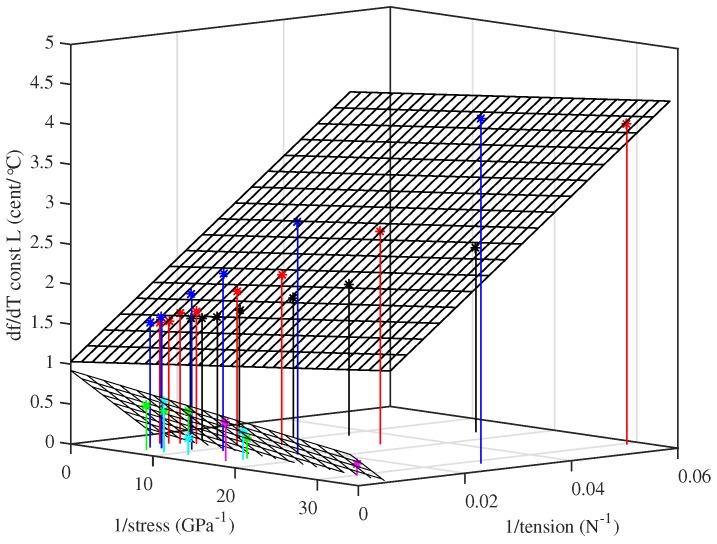
The thermal tuning sensitivity, plotted as a function of the inverse of stress and the inverse of tension, for the strings held at constant length with no tuning adjustments. The *y*-axis shows the rate of change of fundamental frequency $f_{1}$ with temperature *T*. Colours identify the different tested strings (Table 1): black (8:A6), red (14:A5), blue (5:A4), green (23a:A3), cyan (29:E3), magenta (10b:A2).

As for the Young’s modulus results, testing different strings at approximately the same set of stress levels enabled the behaviour in response to other variables to be explored. For any given stress, the tuning sensitivity was found to vary fairly linearly when plotted against either the inverse of the string tension, or the inverse of the cross-sectional area. However, while a single plane could be fitted to the Young’s modulus results across all of the strings tested (Figure 11), separate planes with very different fitted coefficients were required for the thermal tuning sensitivity of the two groups of strings. Figure 14 shows the results using the inverse of the tension as the second variable. It was found that the thermal tuning sensitivity results, for the strings held at constant length, could be described quite well ($r^{2}$ = 0.99 and 0.82 for the lower- and higher-density groups, respectively) by an expression of the form:

(20) $${\left.\frac{df_{¢}}{dT}\right|}_{L}\approx a+\frac{b}{\sigma_{0}}+\frac{c}{F_{0}}\;¢/{}^{\circ}C$$

where a,b and *c* are constant coefficients. The dashed lines shown in Figure 13 were obtained in this way: values of the fitted coefficients are given in the Appendix A. The inverse-stress term is the most important. It may be noted that the notional stress $\sigma_{N}$ and tension $F_{N}$, calculated using the unstretched values for the string bulk and linear density, respectively, could be used in place of $\sigma_{0}$ and $F_{0}$ without significantly affecting the goodness of fit.

**Figure 15:**
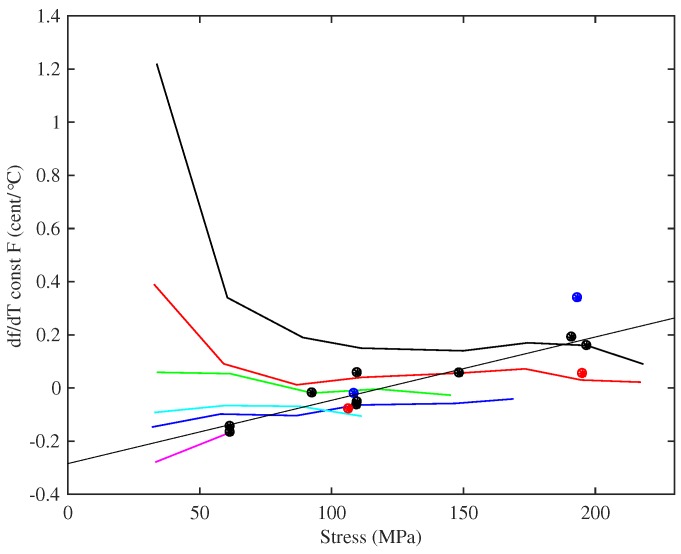
The thermal tuning sensitivity, plotted against stress, for the strings held at constant tension. The *y*-axis shows the rate of change of fundamental frequency $f_{1}$ with temperature *T*. Colours identify the different tested strings (Table 1): black (8:A6), red (14:A5), blue (5:A4), green (23a:A3), cyan (29:E3), magenta (10b:A2). The fitted line ($r^{2}=0.895$) was matched to the data (black circles) for the operating points of the Bowbrand strings tested at the usual humidity levels (around 55–65% RH at 20 °C). Blue circles show Pirastro strings; red circles show tests at low humidity (around 20–26% RH at 20 °C).

Figure 15 shows the thermal tuning sensitivity when the strings were maintained at constant tension. The thermal tuning sensitivities of the strings were significantly reduced, with the sensitivities at the string operating points all smaller than 0.5 $¢{/}^{{}^{\circ}}$C and often less than 0.3 ¢/°C; albeit with the thinner strings going slightly sharp as the temperature rose, while the thicker strings went slightly flat. The effects of thermal changes on the string tension were not entirely removed though. As the temperature rose and the string tightened up (see below), it had to be unwound from the winding shaft to compensate, adding extra mass and increasing the linear density. This usually worked against the thermal variation in the linear density, which was now the dominant effect.

A fairly linear relationship can be seen between the tuning sensitivity at the string operating points and the applied stress. The fitted line in Figure 15 was matched to the data for the Bowbrand strings tested at the usual humidity levels ($r^{2}=0.895$). Noting that, except at the lowest stress levels, the tuning sensitivity of each string showed little variation with stress and recalling the string design scaling rules and the almost linear relationship between the tension and diameter at the operating points, it might be argued that a more fundamental explanatory parameter in this case is the inverse of the string diameter.

### 4.4. Thermal Variations in Tension and Linear Density

**Figure 16:**
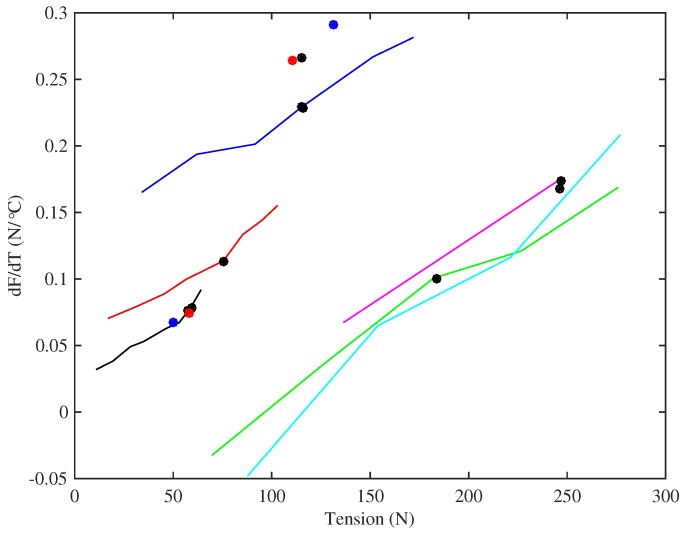
The thermal sensitivity dF/dT of the tension of the strings while held at constant length (with no tuning adjustments). The responses are plotted against the string tension $F_{0}$ at the base temperature (20 °C) to illustrate the strong correlation between these two parameters. Colours identify the different tested strings (Table 1): black (8:A6), red (14:A5), blue (5:A4), green (23a:A3), cyan (29:E3), magenta (10b:A2). Black circles show the results at the expected operating points for the Bowbrand strings tested at the usual humidity levels (around 55–65% RH at 20 °C); blue circles show Pirastro strings; red circles show tests at low humidity (around 20–26% RH at 20 °C).

It is of some interest to dig a little deeper into the data for thermal sensitivity, to see the influence of different factors contributing to the tuning sensitivity. Figure 16 shows the gradients of the lines fitted to the tension versus temperature responses for the strings held at constant length with no tuning adjustments. In nearly every case tested the string tension increased as the temperature rose (provided the string was not heated far enough to cause it to start creeping again). There was a strong correlation between these thermal tension sensitivity values dF/dT and the string tension $F_{0}$ at the base temperature, and Figure 16 uses $F_{0}$ (rather than stress) as the abscissa to illustrate this. The two Pirastro strings and the two Bowbrand strings tested at lower humidity levels show very similar behaviour to the rest of the Bowbrand strings. The responses for the strings tested at multiple target frequencies are fairly linear and nearly parallel, across both groups of strings, each having a gradient of about 0.001/°C.

**Figure 17:**
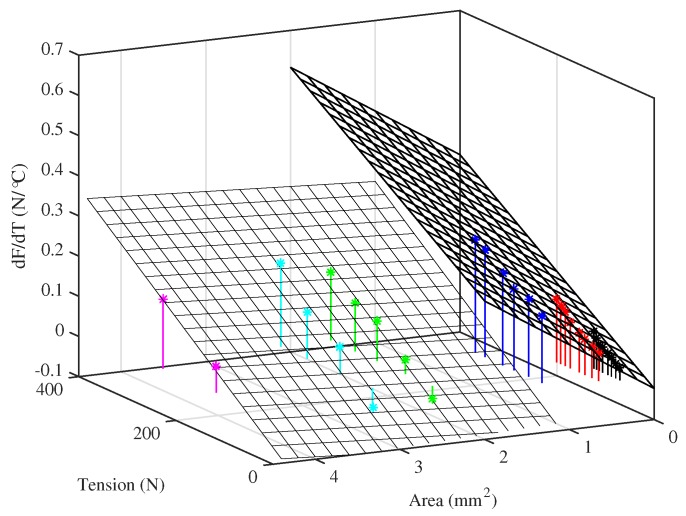
The thermal sensitivity dF/dT of the tension of the strings while held at constant length (with no tuning adjustments) plotted as a function of the string tension $F_{0}$ at the base temperature (20 °C) and the unstretched cross-sectional area $A_{N}$. Colours identify the different tested strings (Table 1): black (8:A6), red (14:A5), blue (5:A4), green (23a:A3), cyan (29:E3), magenta (10b:A2).

Further analysis showed that the thermal tension sensitivity appeared to rise fairly linearly as the string cross-section increased, but with very different gradients for the two groups of strings (Figure 17). Similar behaviour could be observed using the unstretched string diameter or linear density in place of the cross-section. A linear variation with the string cross-section would seem the more intuitively satisfying interpretation, however, as it would correspond directly to the observed correlation between the thermal tuning sensitivity and the inverse of the applied stress (see Equations (10) and (20)).

**Figure 18:**
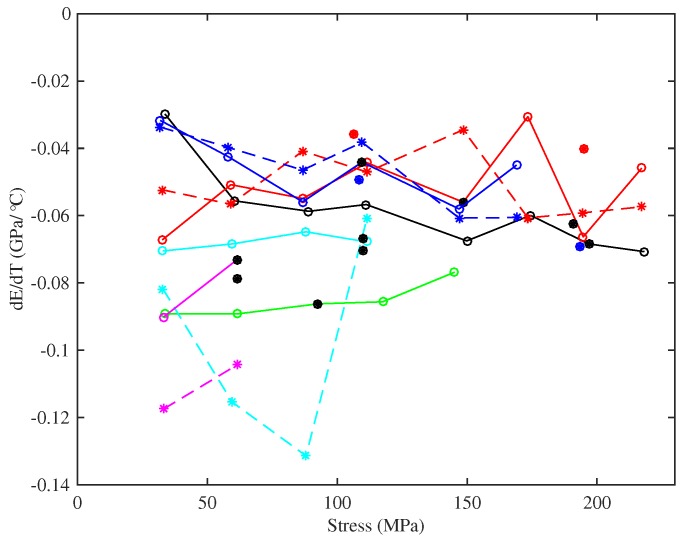
The rates of thermal variation in Young’s modulus, about the base temperature of 20 °C, plotted against the applied stress. The solid lines show the results obtained using tension modulation, while the dashed lines provide a comparison with the values derived from the string bending stiffness. The latter data were only available for the last four strings tested, after the harmonic analysis capability had been added into the test rig control program. Colours identify the different tested strings (Table 1): black (8:A6), red (14:A5), blue (5:A4), green (23a:A3), cyan (29:E3), magenta (10b:A2). Black circles show the results at the expected operating points for the Bowbrand strings tested at the usual humidity levels (around 55–65% RH at 20 °C); blue circles show Pirastro strings; red circles show tests at low humidity (around 20–26% RH at 20 °C).

Figure 18 shows the gradients of lines fitted to Young’s modulus versus temperature responses. There is significant scatter in the responses, possibly due to the difficulty in extracting an accurate gradient measure in some cases, but the broad conclusion is that the rate of thermal variation in the Young’s modulus appeared to stay fairly constant as the applied stress increased; while, as seen earlier, the base temperature Young’s modulus increased steadily with stress. In all cases, the values were negative: the Young’s modulus falls as the temperature increases, which would make the string easier to stretch, hence tending to reduce the tension and make the string go flat. This negative gradient has already been seen in the DMA tests shown in Figure 8 and is a familiar effect for all polymers [20]. The thermal sensitivities of the strings tested at lower humidity levels were slightly lower than for other strings of the same gauges, but the differences were small compared to the range of variation seen in the strings tested at multiple target frequencies. The two Pirastro strings displayed rates of thermal variation in Young’s modulus that were broadly similar to the values obtained from the equivalent Bowbrand strings tested at the usual humidity levels.

Figure 18 includes the thermal variation gradients for the Young’s modulus values obtained from the string bending stiffness for the last four strings tested (these measurements were not taken for the earlier strings). The results for the thicker E3 and A2 strings show a greater degree of variation than those for the thinner A5 and A4 strings. This was due to poorer performance of the automated harmonic identification and curve fitting algorithm used to extract the bending stiffness values (Section 3.6). For the thinner strings, at least, the thermal sensitivities of the two measures of Young’s modulus are notably similar given the consistent difference in the corresponding base-temperature Young’s modulus values. Recalling the earlier remarks that the quantities being measured were more strictly $\left(1/A_{0}\right)d\left(AE_{T}\right)/dT$ and $\left(1/I_{0}\right)d\left(IE_{B}\right)/dT$ rather than dE/dT per se, this suggests that any thermal variation in the string diameter was perhaps relatively small.

**Figure 19:**
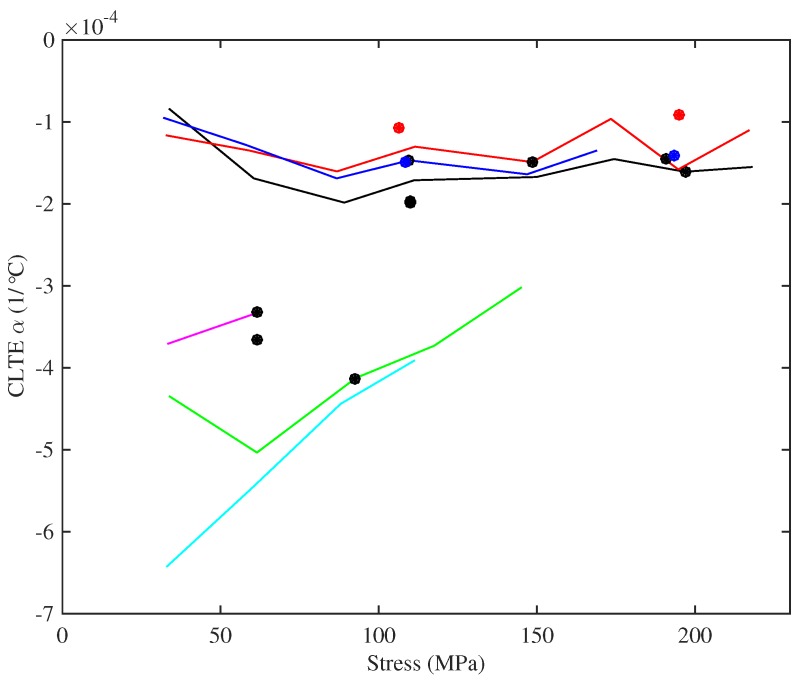
The coefficient of linear thermal expansion (CLTE) along the strings, plotted against the applied stress. The CLTE values were derived from the thermal tension sensitivity, in the absence of any tuning adjustments, and the thermal variation in Young’s modulus. Colours identify the different tested strings (Table 1): black (8:A6), red (14:A5), blue (5:A4), green (23a:A3), cyan (29:E3), magenta (10b:A2). Black circles show the results at the expected operating points for the Bowbrand strings tested at the usual humidity levels (around 55–65% RH at 20 °C); blue circles show Pirastro strings; red circles show tests at low humidity (around 20–26% RH at 20 °C).

Figure 19 shows the CLTE values obtained for the various strings, plotted against the applied stress. The CLTE values were derived using Equation (16), from the constant-length thermal tension sensitivity and the thermal variation in Young’s modulus. Again, there is significant scatter in the responses for the strings tested at multiple frequencies, but overall, the CLTE appeared to remain fairly constant as the stress was changed, at least for the lower-density set of strings.

In all cases, the CLTE was negative, indicating that the strings were trying to contract longitudinally as they were heated, which would tend to increase the tension and make the strings go sharp. This is a well-known effect, usually explained in terms of entropy [22]. The nylon molecules can adopt more bent configurations than straightened configurations, so when heat is applied and energy added, the distribution of configurations changes with more molecules taking on bent configurations (or at least endeavouring to do so). As with the thermal variation in Young’s modulus, the CLTE values were slightly smaller for the strings tested at lower humidity levels, but again, the differences were not great; the Pirastro strings again showed values broadly similar to the equivalent Bowbrand strings.

It is worth noting that the CLTE values obtained for the strings were nearly two orders of magnitude larger than the expected range of values for the hardwood baseboard (Section 3.1). Indeed, a steel base could probably have been used quite satisfactorily without requiring any compensation for its thermal expansion.

**Figure 20:**
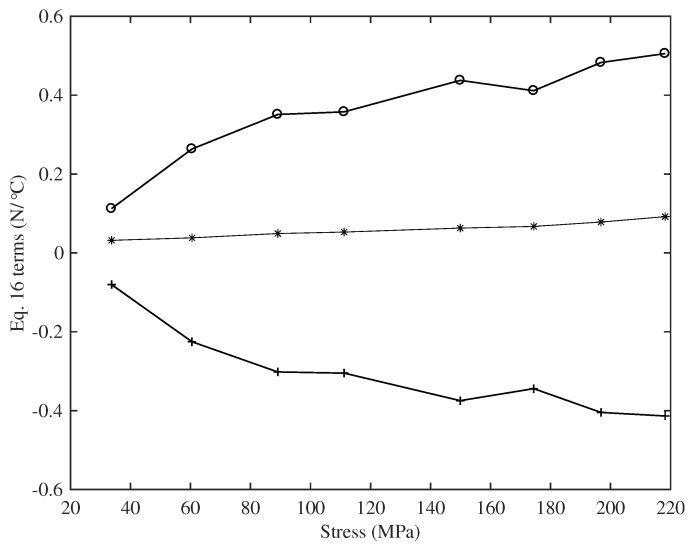
A comparison of the different terms of Equation (16) for one of the nylon A6 strings (String 8). The lower line (+) shows $\left(F_{0}/A_{0}E_{0}\right)d\left(AE\right)/dT$; the upper line (o) shows $-A_{0}E_{0}\alpha$; and the middle line (*) gives the combined term dF/dT taken from the gradient of the tension versus temperature response of the string held at constant length.

The two thermal effects, the reduction in the Young’s modulus and the thermal contraction of the string as the temperature rises, work against each other, with the balance between them determining the overall variation in the string tension. This balance between the two effects was, however, quite close, as is illustrated in Figure 20: the two terms on the right side of Equation (16) were almost equal (and opposite) and around an order of magnitude greater than the difference between them. Consequently, any measurement errors in the thermal variation of Young’s modulus were directly reflected into similar variations and errors in the CLTE values.

**Figure 21:**
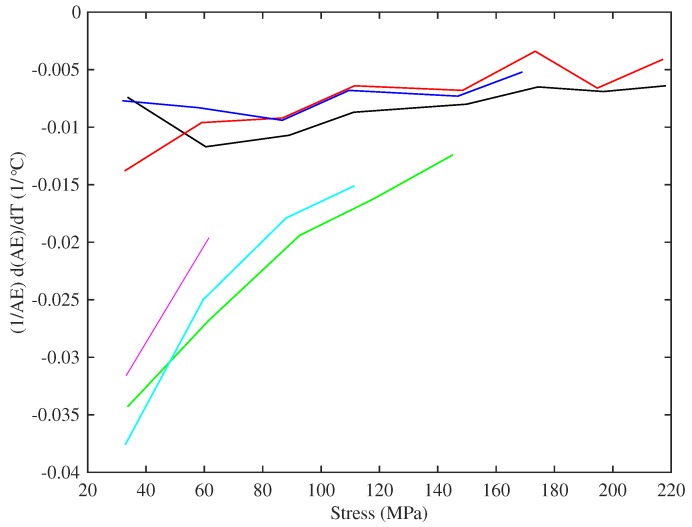
$\left(1/A_{0}E_{0}\right)d\left(AE\right)/dT$ plotted against the applied stress. Colours identify the different tested strings (Table 1): black (8:A6), red (14:A5), blue (5:A4), green (23a:A3), cyan (29:E3), magenta (10b:A2).

From Equation (16) and the rather linear responses shown in Figure 16, it might be expected that the $\left(1/A_{0}E_{0}\right)d\left(AE\right)/dT$ and $A_{0}E_{0}\alpha$ terms should both be constant for each string. Figure 21, which shows $\left(1/A_{0}E_{0}\right)d\left(AE\right)/dT$ plotted against stress, demonstrates that this is not the case. The behaviour shown in Figure 16 might therefore suggest some underlying connection between the thermal variation in Young’s modulus and the CLTE, common to both groups of strings.

**Figure 22:**
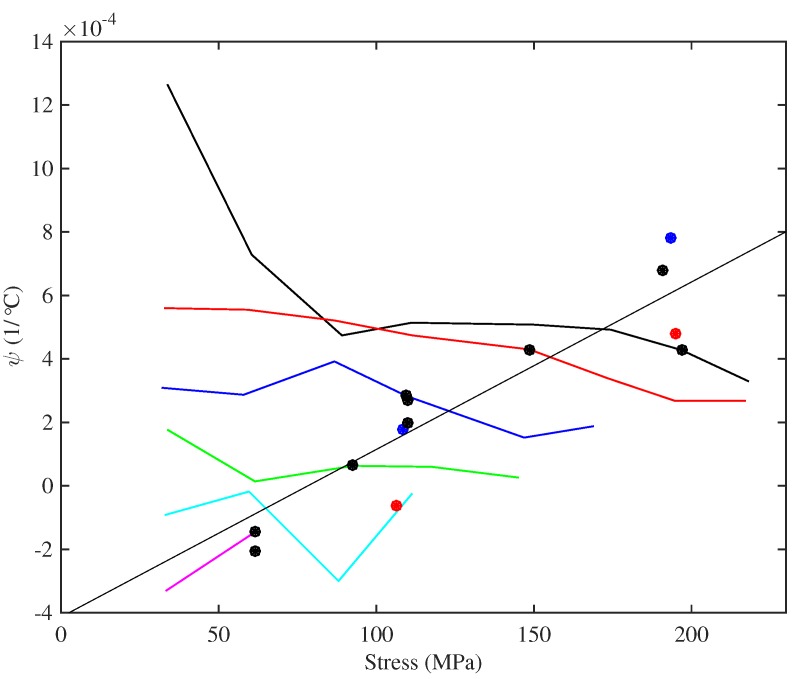
The thermal variation coefficient ψ for the string linear density, plotted against the applied stress. Colours identify the different tested strings (Table 1): black (8:A6), red (14:A5), blue (5:A4), green (23a:A3), cyan (29:E3), magenta (10b:A2). The fitted line ($r^{2}=0.858$) was matched to the data (black circles) for the operating points of the Bowbrand strings tested at the usual humidity levels (around 55–65% RH at 20°C). Blue circles show Pirastro strings; red circles show tests at low humidity (around 20–26% RH at 20°C).

The final measurement, shown in Figure 22, is of the thermal variation coefficient ψ for the string linear density (see Equation (11)). It is plotted against the applied stress. For the strings tested at multiple target frequencies, the thermal sensitivity of the string linear density appeared to be largely independent of the applied stress; at least at the higher stress levels. However, if only the string operating points are considered, a different pattern is seen: the results suggest a steady and almost linear increase in ψ with stress. The fitted line in Figure 22 ($r^{2}=0.858$) was matched to the data for the Bowbrand strings tested at the usual humidity levels (around 55–65% RH at 20°C). As for the thermal tuning sensitivity of the strings held at constant tension, the best explanatory parameter might actually be the inverse of the string diameter. There are some striking similarities between Figure 15 and Figure 22, consistent with the thermal tuning sensitivity at constant tension being determined primarily by variations in the string linear density.

It is interesting to note that while most of the strings had positive values for ψ, indicating that in those cases, the strings were losing mass (perhaps by drying out) as the temperature increased, the thickest strings had negative values, indicating they gained mass as the temperature rose. The two Pirastro strings appeared to follow the same trend as the Bowbrand strings, while the strings tested at lower humidity levels (around 20–26% RH at 20 °C) had lower values of ψ than the comparable strings tested at the usual humidity levels. The latter result is, perhaps, unsurprising, since the thermal variation in both the absolute and relative humidity was much lower during the tests at the lower humidity levels.

In every case, the value of ψ was smaller, and often much smaller, than $\left(1/F_{0}\right)dF/dT$, indicating that the tuning behaviour of the strings held at constant length (Equation (10)) was dominated by the thermal variation in the string tension. In most cases, there was a reduction in the string linear density as the temperature rose, which contributed to the string sharpening, but this effect was small compared to the tension variation.

## 5. Discussion and Conclusions

### 5.1. Material Variations

This study has revealed a complicated pattern of mechanical behaviour in monofilament nylon harp strings, even though attention has been focussed solely on the long-term behaviour of well-settled strings: another layer of complication appears when time-dependent phenomena during the settling process are examined, but that is a topic for future work. The first striking thing revealed by the tests reported here is that the strings were not all the same. Although the tested strings were commercially marketed as a homogeneous set, the properties fell into two (or possibly more) distinct groups. The thinner strings had lower density than the thicker strings, and this density difference turned out to correlate with very different stress-strain behaviour and also with different response to temperature fluctuations. Whether the difference arose from chemistry or from an effect of manufacturing process could not be determined by the test methods available in this study.

For both groups, many of the long-term mechanical properties of the stretched nylon strings differed markedly from those published for bulk nylon, presumably as a result of molecular alignment caused by the drawing process during the string manufacture and subsequent stretching. These differences appeared both in the Young’s modulus and in the response to thermal changes, as will now be detailed.

### 5.2. Stretching Behaviour

The Young’s modulus of bulk nylon at 50% RH is quoted in the literature in the range of 1.5–2 GPa [6]. This study reveals a far more complicated picture, which cannot be encapsulated in any single number. Young’s modulus has been shown to be a strong function of the testing frequency, as is not entirely surprising for a viscoelastic material. It is also a strong function of the stress or strain state of the string: and it should be noted that the conventional stringing pattern on an instrument like the harp embodies large variations of stress, with relatively low stress in the lower sounding strings, while the top strings are close to their ultimate breaking stress.

Values of Young’s modulus have been measured in this study by a variety of methods, between them covering a very wide range of frequency (or time-scale). On the longest time-scale, the stress-strain curves of strings have been measured on a scale of weeks, and their slope gives the Young’s modulus near the DClimit. On the same test rig, tension modulation on a scale of minutes has been used. Finally, the bending stiffness of the strings was measured from its effect on the pattern of overtone frequencies. An excellent fit to the predicted theoretical pattern was found, and this allows a calculation of Young’s modulus in the frequency range of the overtones tested and, thus, on a millisecond time-scale. In addition, some unstretched samples of strings were tested in a DMA to fill in a range of intermediate frequencies. These results all seem compatible, in that a rather smooth trend of Young’s modulus with frequency can be assembled by combining them: see Figure 9. Over this frequency range, the value of Young’s modulus changed by roughly an order of magnitude, with the highest values at the highest frequencies, as expected.

For a given test method, the modulus also changed significantly with stress. As an example, the tension-modulation results for one particular string increased steadily with the applied stress from about 3 GPa (at a stress of 30 MPa) to nearly 11 GPa (at 220 MPa). All of these values are significantly higher than the textbook value for bulk nylon. The marked difference in the behaviour of the stretched strings, when compared to the bulk material, was presumably a consequence of molecular alignment within the drawn and stretched strings, progressively stiffening as the nylon molecules were straightened (note that this is a quite different physical phenomenon from strain hardening in metals, driven by the movement and pinning of dislocations).

Young’s modulus of the strings also varied with the string density, decreasing as the density increased. This variation was less significant than the change with the applied stress, but appeared to be fairly linear across the full range of string densities tested (Figure 11). It is not known why the Young’s modulus should decrease as the string density rises, but the fact that the behaviour appeared common across both groups of strings, despite the marked differences in their other properties, perhaps suggests an effect fundamental to the nylons.

The two main groups of strings showed very different long-term stress-strain behaviour: see Figure 6. The DMA tests gave a clue about the origin of this contrast: as well as testing with a frequency sweep, it was possible to scan a range of temperatures at fixed frequency. The results (see Figure 8 for an example) showed that the two groups of strings had very different glass transition temperatures, and frequency dependence and temperature dependence are known to be very strongly linked in polymer viscoelasticity.

### 5.3. Responses to Temperature Changes

Direct empirical measurements were made of the sensitivity of the string’s tuning to temperature changes. Two cases were studied: the unadjusted string, held at constant length, and the case in which tension was held constant by a feedback controller. In the former case, directly relevant to a musician, quite high sensitivity to temperature was seen with a very strong correlation with the inverse of stress. The detailed pattern was consistent within each group of strings, but very different between the two groups (see Figure 13): they even showed opposite signs of the inverse-stress effect. When tension was held constant, the sensitivity was greatly reduced, but did not disappear entirely.

The thermal sensitivity of tuning has its origin in the sensitivities of the various contributory factors, and these have also been explored in this study. The thermal variation in Young’s modulus is one area in which there does seem to be a fairly close agreement between the values obtained for the tested strings, being in the range of -0.03 to -0.09 GPa/°C, and the published values available for bulk nylon, which range between about -0.03 and -0.05 GPa/°C [7,8]. The longitudinal thermal expansion behaviour, however, is completely different: bulk nylon expands as it is heated, with a CLTE of around 80–$100\times 10^{-6}/{}^{\circ}$C [6], whereas the tested strings all attempted to contract. This difference stems from an entropic effect associated with the molecular orientation within the strings. Both the rate of thermal variation in Young’s modulus and the longitudinal CLTE appeared largely unaffected by changes in the applied stress. They combine to produce the overall effect of temperature changes on the string tension, and hence on tuning, but they act in opposite senses with magnitudes that are almost equal, so they almost cancel each other out.

Changes in the string linear density were found to be relatively small, making only a minor contribution. The effects of humidity changes, while not studied extensively, also appear to be small enough that they can, for practical purposes, be ignored.

The finding that some aspects of the thermal behaviour were common across both groups of strings, while others were markedly different, might be hinting at some fundamental distinction: perhaps the former are associated with intra-molecular effects common across the nylons, while the latter are governed by inter-molecular forces more affected by differences in chemical make-up. Those behaviours determined by intra-molecular effects might then also be expected to display a high level of interdependency, as hinted at by the thermal tension sensitivity behaviour shown in Figure 16.

### 5.4. Implications for Tuning Compensators

Correlation studies of the detailed results reported here reveal some intriguing trends, for many of which the authors have, at present, no explanation. A variety of regression fits have been presented, summarised in the Appendix A, since these may be of value in any detailed attempts to implement automated tuning compensation for nylon strings.

Having developed simple and apparently quite accurate expressions for predicting both Young’s modulus and the thermal tuning sensitivity of an unadjusted nylon string, it is tempting to think that such a model could be combined with a thermometer to create a tuning control system able to predict the likely tuning deviation in response to a change in temperature and the required string winding adjustment to correct it. On the test rig, it was possible to go further than this by maintaining the string at the required frequency, while cycling the temperature and recording the required string length adjustments. Lines and curves could then be fitted to the length adjustment versus temperature responses and used to provide tuning compensation functions, which directly predicted the length adjustment required to compensate for a given change in temperature. Tests showed that this approach typically worked well over the short term, while the temperature was increasing or decreasing, but that the compensation tended to drift over the long term, especially following temperature reversals.

Any tuning compensation approach that attempts to predict the required winding length adjustments based on changes in temperature or humidity is likely to suffer from a number of weaknesses. Essentially, only the string extension is being controlled; any unexpected changes in the string length or stiffness, and in particular any string creep, will go uncorrected. More fundamentally, such an approach is effectively open-loop, so any adjustment errors (which will always be there) can be expected to accumulate over time. Having said that, such an approach may still prove effective when used in conjunction with a closed-loop tuning system requiring the strings to be plucked to give a direct pitch measurement; the latter could be used to retune the instrument during breaks in the performance, with the open-loop compensator maintaining the string tuning during the intervening periods.

Given the findings that changes in the string linear density, and indeed in the ambient humidity, could largely be ignored, it would be expected that the string tuning could be adequately controlled through monitoring the string tension; effectively providing a closed-loop compensator able to take account of changes in both the string stiffness and its extension. This indeed proved to be the case. On the test rig, it was also possible to measure the tension adjustments required to maintain the string frequency and then extract a tuning compensation function for adjusting the tension as a function of temperature. This approach gave very good, stable results, but would require an accurate temperature-compensated tension sensor to be fitted to every string on an instrument.

In practice, maintaining nylon strings of the type tested here at constant tension would probably be more than adequate; with a sensor able to detect a change in tension likely to be considerably less expensive that one able to provide an absolute tension measurement. This approach also opens up the possibility of a mechanical compensator based on some spring and lever arrangement, but there may be concerns regarding how such a device, being always active, might affect the string feel and any string pitch distortion or other transient effects when the string is plucked.

This study has ignored the effects of any environmental changes on the instrument body. On the harp, the soundboard in particular, even though usually varnished on both sides, may be expected to change in mass and/or stiffness in response to temperature and humidity changes. The changes in string behaviour have been shown here to be significant in themselves, but further tests with strings mounted on an instrument would be needed to determine the relative contribution of the frame and soundboard and the effectiveness of any chosen tuning compensation approach.

As a final note of caution: because the required string length adjustments are so small, static friction between the string and the bridge pin adjacent to the winding peg must be considered. In an electronic system, the shaft of the bridge pin could be rotated during any adjustment to break the static friction, but this would be more difficult to achieve in a mechanical solution.

## Figures and Tables

**Table 1:** The set of nylon harp strings studied, showing the unstretched string parameters, the target fundamental frequencies for testing and the plot styles used in the responses shown in the figures. The number in the first column provides a unique reference for each test string or string section. It can be used to cross-reference with the summary dataset submitted with this paper and the larger dataset available at https://doi.org/10.17863/CAM.9018. Strings 12 and 13 were from the Pirastro “Nycor Concert Harp” range. The other strings were all from the Bowbrand “Pedal Nylon” range. The nine test frequencies marked with an asterisk correspond to the expected operating points on the harp. These points are included in some of the figures as black circles.

No.	Note	Diameter (mm)	Bulk Density (kg/m${}^{3}$)	Target Test Frequencies (Hz)	Plot Style	Comments
1	A4 (19)	1.193	1067	323	Red circle	Tested only at low humidity
6	A6 (5)	0.661	1089	429 *	Red circle (low hum.)	Tested at both humidity levels
2	A4 (19)	1.199	1068	323 *		
3	A4 (19)	1.194	1080	323 *		
10a	A2 (33)	2.362	1134	235 *		Higher density
13	A6 (5)	0.613	1074	429	Blue circle	
12	A4 (19)	1.280	1070	323	Blue circle	
8	A6 (5)	0.658	1097	174, 235, 287, 323, 375, 403, 429 *, 453	Black line	
14	A5 (12)	0.841	1072	174, 235, 287, 323, 375 *, 403, 429, 453	Red line	
5	A4 (19)	1.196	1076	174, 235, 287, 323 *, 375, 403	Blue line	
23a	A3 (26)	1.680	1151	174, 235, 287 *, 323, 360	Green line	Higher density
29	E3 (29)	1.894	1159	174, 235, 287, 323	Cyan line	Higher density
10b	A2 (33)	2.362	1134	174, 235 *	Magenta line	Higher density

## Supplementary Materials

The following are available online at www.mdpi.com/1996-1944/10/5/497/s1: spreadsheets “NLTP master data file”, “NLTP data for Figure 2” and “NLTP data for Figure 3”. A much larger comprehensive dataset is available at https://doi.org/10.17863/CAM.9018.

## Abbreviations

The following abbreviations are used in this manuscript:

ADC	Analogue to digital converter
CLTE	Coefficient of linear thermal expansion
DMA	Dynamic mechanical analyser
RH	Relative humidity

## Appendix A. Fitted Expressions

Table A1 lists the different fitted expressions discussed above, together with the fitted coefficients for the two groups of strings. It should be noted that these expressions are unlikely to apply to other string materials, and may also not apply to dyed nylon strings (such as the red and black or blue strings used to identify the C and F strings on concert and lever harps), or to nylon strings that have not been centreless ground to remove the outer skin which results from the manufacturing extrusion process.

**Table A1:** Summary of the fitted expressions.

Parameter	Fitted Expression	Coefficients and $r^{2}$	
		Lower Density Strings (6, 8, 14, 2, 3, 5)	Higher Density Strings (23a, 29, 10a, 10b)
Young’s modulus from tension modulation (GPa)	$E_{T0}\approx a+b.\sigma_{N}+c.\rho_{N}$	a:32.0, b:0.0353, c:-0.0269, $r^{2}:0.983$	
Young’s modulus from harmonic analysis (GPa)	$E_{B0}\approx a+b.\sigma_{N}$	a:4.53, b:0.0389, $r^{2}:0.957$	
Thermal tension sensitivity, constant length (N/°C)	${\left.\frac{dF}{dT}\right|}_{L}\approx a+b.F_{N}+c.A_{N}$	a:-0.0256 b:0.000784 c:0.144 $r^{2}:0.989$	a:-0.143 b:0.000868 c:0.0166 $r^{2}:0.956$
Thermal tuning sensitivity, constant length ($¢{/}^{\circ }$C)	${\left.\frac{df_{¢}}{dT}\right|}_{L}\approx a+\frac{b}{\sigma_{N}}+\frac{c}{F_{N}}$	a:1.067 b:115 c:-11.3 $r^{2}:0.990$	a:0.890 b:-14.7 c:-67.1 $r^{2}:0.803$
